# Characterization of three *Stenotrophomonas* strains isolated from different ecosystems and proposal of *Stenotrophomonas mori* sp. nov. and *Stenotrophomonas lacuserhaii* sp. nov.

**DOI:** 10.3389/fmicb.2022.1056762

**Published:** 2022-12-15

**Authors:** Yang Deng, Xue-Fei Han, Zhu-Ming Jiang, Li-Yan Yu, Yong Li, Yu-Qin Zhang

**Affiliations:** ^1^Institute of Medicinal Biotechnology, Chinese Academy of Medical Sciences and Peking Union Medical College, Beijing, China; ^2^State Key Laboratory of Dao-di Herb, Beijing, China; ^3^Faculty of Geosciences and Environmental Engineering, Southwest Jiaotong University, Chengdu, China

**Keywords:** *Stenotrophomonas mori*, *Stenotrophomonas lacuserhaii*, genome, ANI, dDDH, IAA

## Abstract

*Stenotrophomonas* spp. have primarily been reported as non-pathogenic, plant-probiotic bacteria, despite the presence of some opportunistic human pathogens in the genus. Here, three Gram-stain negative, rod-shaped, non-spore-forming bacteria, designated as strains CPCC 101365^T^, CPCC 101269^T^, and CPCC 101426 were isolated from surface-sterilized medicinal plant roots of a mulberry plant in Chuxiong of the Yunnan Province, freshwater from Erhai Lake in the Yunnan Province, and sandy soils in the Badain Jaran desert in Inner Mongolia Autonomous Region, China, respectively. The 16S rRNA gene sequences analysis of these isolates in comparison with sequences from the GenBank database indicated that they belong to the genus *Stenotrophomonas*, with nucleotide similarities of 96.52–99.92% to identified *Stenotrophomonas* members. Phylogenetic analysis based on 16S rRNA gene and genome sequences confirmed that the isolates are members of the genus *Stenotrophomonas*. Values for genomic average nucleotide identity (ANI; <95%) and digital DNA–DNA hybridization (dDDH; < 70%) indicated that strains CPCC 101365^T^ and CPCC 101269^T^ were well-differentiated from validly described *Stenotrophomonas* species, while strain CPCC 101426 shared high ANI (97.7%) and dDDH (78.3%) identity with its closest phylogenetic neighbor, *Stenotrophomonas koreensis* JCM 13256^T^. The three genomes were approximately 3.1–4.0 Mbp in size and their G + C content ranged in 66.2–70.2%, with values slightly differing between CPCC 101365^T^ (3.4 Mbp; 70.2%), CPCC 101269^T^ (4.0 Mbp; 66.4%), and CPCC 101426 (3.1 Mbp; 66.2%). Genes encoding enzymes involved in the biosynthesis of *indole-3-acetic acid* (IAA) and siderophores were identified in the genomes of the three isolates, suggesting that these strains might serve roles as plant-growth promoting microorganisms. The polar lipid fractions of the three isolates primarily comprised diphosphatidylglycerol (DPG), phosphatidylglycerol (PG), and phosphatidylethanolamine (PE). The predominant cellular fatty acid was iso-C_15_: _0_, with moderate amounts of antesio-C_15: 0_, iso-C_11: 0_, iso C_17: 1_*ɷ*9c/C_16: 0_ 10-methyl, iso-C_14: 0_, and C_16: 1_*ɷ*7*c*/C_16: 1_*ɷ*6*c*. These results indicated that polyphasic characteristics of strains CPCC 101365^T^ and CPCC 101269^T^ differed from other identified *Stenotrophomonas* species and that strain CPCC 101426 was affiliated with the species *Stenotrophomonas koreensis*. Accordingly, two novel species of the genus *Stenotrophomonas* were consequently proposed, corresponding to *Stenotrophomonas mori* sp. nov. (type strain CPCC 101365^T^ = DY006^T^ = KCTC 82900^T^) and *Stenotrophomonas lacuserhaii* sp. nov. (type strain CPCC 101269^T^ = K32^T^ = KCTC 82901^T^).

**Highlights:**

Members of the genus *Stenotrophomonas*, and particularly *Stenotrophomonas maltophilia*, are opportunistic human pathogens, but not enough research has evaluated the identification of environmental *Stenotrophomonas* spp. However, most *Stenotrophomonas* spp. serves as plant-probiotic bacteria.

In this study, we obtained and characterized three *Stenotrophomonas* strains from different ecosystems. Based on phenotypic differences, chemotaxonomic properties, ANI and dDDH identity values, and phylogenetic analyses, two novel *Stenotrophomonas* species are proposed for the strains identified here. The encoding genes related to plant-growth promotion in the genomes of the newly recovered *Stenotrophomonas* spp. were retrieved. Follow-on experiments confirmed that these strains produced the important plant hormone IAA. Thus, these *Stenotrophomonas* spp. could considerably contribute to shaping and maintaining ecological stability in plant-associated environments, particularly while acting as plant-probiotic microorganisms.

## Introduction

The genus *Stenotrophomonas* was formally proposed by Palleroni and Bradbury (1993), with *Stenotrophomonas maltophilia* as the type species based on morphological, physiological, and biochemical comparisons to *Xanthomonas maltophilia*, and other *Xanthomonas* spp. (Hugh and Leifson, 1953). Prior to the valid description of the name *Stenotrophomonas*, *S*. *maltophilia* was investigated and independently described as *Bacterium booker, Pseudomonas maltophilia,* and *Xanthomonas maltophilia* in several studies (Hugh and Ryschenkow, 1961; Hugh and Leifson, 1963; Komagata et al., 1974; Ikemoto et al., 1980; Swings et al., 1983; Palleroni and Bradbury, 1993). The species *S*. *maltophilia* comprises a group of opportunistic pathogens with low virulence that causes a variety of human infections, is also associated with plant rhizospheres, and contributes to sulfur and nitrogen biogeochemical cycling. A total of 16 different *Stenotrophomonas* species have been previously identified from various niches,^1^ including upflow anaerobic sludge blanket reactors (Assih et al., 2002), bentonite formations (Sanchez-Castro et al., 2017), sewage (Kaparullina et al., 2009; Lee et al., 2011), ginseng fields (Kim et al., 2010), compost (Yang et al., 2006), surfaces with food contact (Weber et al., 2018), as a contaminant of stone chamber interiors (Handa et al., 2016), in ammonia-supplied biofilters (Finkmann et al., 2000), in the rhizosphere of oilseed rape, geocaulsopheres (tuber) of potato (Wolf et al., 2002), infected human body (Coenye et al., 2004), and other habitats. Additionally, *Stenotrophomonas maltophilia* complex (Smc) was recognized as high levels of phenotypic and genotypic diversity (Patil et al., 2018). *Stenotrophomonas maltophilia* is the only identified human opportunistic pathogenic species of the genus and most *Stenotrophomonas* spp. exhibit functions related to plant growth promotion, improving bioremediation, synthesizing nanoparticles, and improving resistance to multi-drug resistant pathogens (Nakayama et al., 1999; Suckstorff and Berg, 2003; Dubey and Fulekar, 2012; Oves et al., 2013). In this study, three *Stenotrophomonas* strains were characterized and reported that were isolated from a mulberry plant sample in Chuxiong, a water sample from Erhai lake, and a gravel soil sample from the Badain Jaran desert. Phylogenetic analysis, genomic comparisons, and comparisons of chemotaxonomic and phenotypic characteristics were used to classify one strain as *S*. *koreensis,* and propose that the other two strains represent novel *Stenotrophomonas* species.

## Materials and methods

### Strain acquisition

Strain CPCC 101365^T^ was isolated from a mulberry root sample collected from Chuxiong (25°02′ N, 101°54′ E) in the Yunnan Province of southwestern China. The plant root sample was slowly cleaned using flowing tap water to remove adhered clay particles on the surface. The root sample was then dipped in an ultrasonic washing instrument (at 40 W for 5 min) to further remove root contaminants. After air-drying the surface moisture, the sample was subjected to five-step surface sterilization (Qin et al., 2009). Briefly, in the first step, the sample was submersed in solution A (5% NaOCl), while maintaining gentle shaking for 10 min. In the second step, the sample was transferred into solution B (2.5% Na_2_S_2_O_3_) and maintained for 10 min. In the third step, the sample was submersed in solution C (75% ethanol) for 5 min. In the fourth step, the sterilized sample was washed using sterile water three times to remove remaining ethanol. In the fifth step, the sample was rinsed with 10% NaHCO_3_ for 10 min. The surface-sterilized samples were placed on aseptic paper in a sterile vertical flow clean bench overnight until their moisture was dried. Besides NaOCl and ethanol were used as the surface disinfectants here, the solution B (2.5% Na2S_2_O_3_) served as the antichlor to remove the residual, and NaHCO_3_ served as buffering agent to stabilize the pH value. Samples were then aseptically ground into small fragments and the pieces were directly placed on humic acid agar medium containing 1.0 g L^−1^ humic acid, 1.0 g L^−1^ asparagine, 0.01 g L^−1^ FeSO_4_·7H_2_O, 0.5 g L^−1^ Na_2_HPO_4_·12H_2_O, 1.7 g L^−1^ KCl, 0.02 g L^−1^ CaCO_3_, and 15.0 g L^−1^ agar (pH 7.2). After incubation at 28°C for 3 weeks, individual colonies were picked and streaked onto newly prepared PYG medium plates (containing 3.0 g L^−1^ peptone, 5.0 g L^−1^ yeast extract, 10.0 ml glycerol, 1.25 g L^−1^ betaine hydrochloride, 1.25 g L^−1^ sodium pyruvate, 15.0 g L^−1^ agar, and pH 7.2) to obtain pure cultures.

Strain CPCC 101269^T^ was isolated from a water sample collected from Erhai Lake (25°40′ N, 100°12′ E), which is a freshwater reservoir in Dali, within the Yunnan Province of southwestern China. The water sample (500 ml) was filtered through 3.0 μm pore size filters to exclude most cyanobacterial colonies and other small particles, followed by filtering water to concentrate on a 0.22 μm pore-size filter. The filter was subsequently sectioned into small pieces and suspended into 5 ml of a sterile NaCl solution (0.85%). Approximately 0.2 ml of the solution was spread onto PYG medium for cultivation. After incubation at 28°C for 3 weeks, individual bacterial colonies were picked and streaked onto newly prepared PYG slants for purification, as evinced by the appearance of uniform colonies.

Strain CPCC 101426 was isolated from a gravel soil sample collected from the Badain Jaran desert (39°24′ N, 102°31′ E) in China. About 2 g of each sample was suspended in 18 ml of a 0.85% (w/v) NaCl solution, then 200 μl of the 10^−4^ diluted soil suspension was spread onto humic acid agar medium. After incubating for 3 weeks at 28°C, visible colonies were picked and streaked onto PYG medium and again incubated at 28°C to obtain isolated colonies. The purified isolates were maintained in glycerol suspensions (20%, v/v) at −80°C.

### Phylogenetic analysis

The genomic DNAs from these strains were prepared and 16S rRNA genes were amplified and sequenced according to Li et al. (2007). The 16S rRNA gene sequences of the isolates were compared with publicly available sequences in the EzBioCloud^2^ platform to determine the approximate phylogenetic affiliations of the strains (Yoon et al., 2017a). Multiple sequence alignments of the most closely related taxa and calculations of sequence similarity levels were conducted using MEGA version X (Kumar et al., 2018). A phylogenetic tree was then inferred using neighbor-joining methods (Saitou and Nei, 1987) with K values (Kimura, 1979; Kimura, 1980) and complete deletion gaps. Maximum Parsimony (Kluge and Farris, 1969) and Maximum Likelihood (Felsenstein, 1981) phylogenetic methods were also used to evaluate the phylogenetic affiliations of the strains. The topologies of the resultant phylogenetic trees were evaluated using bootstrap analysis with 1,000 replicates (Felsenstein, 1985).

### Genome sequencing and the basic analysis

The genomes of these strains were sequenced using DNBSEQ platform at the Beijing Genomics Institute (Beijing, China). Genomic DNA was sheared randomly to construct three read libraries with lengths of 300 bp by a Bioruptor ultrasonicator (Diagenode, Denville, NJ, United States) and physico-chemical methods. The paired-end fragment libraries were sequenced. Raw reads of low quality from paired-end sequencing (those with consecutive bases covered by fewer than five reads) were discarded. The sequenced reads were assembled using SOAPdenovov1.05 software. The completeness and contamination of the genomes were estimated by the CheckM pipeline. And the potential contamination was also detected by ContEst16S using 16S rRNA genes from genome assemblies.^3^ Both methods were complementary in the detection of contamination. Digital DNA–DNA hybridization (dDDH) and average nucleotide identity (ANI) values between these strains and other related strains were calculated using the Genome-to-Genome Distance Calculator (GGDC, version 3.0; http://ggdc.dsmz.de/ggdc.php; Auch et al., 2010) and with the ezbiocloud platform (Yoon et al., 2017b), respectively. A whole genome phylogeny was generated using the TYGS server (http://tygs.dsmz.de; Meier-Kolthoff and Göker, 2019). The Genome BLAST Distance Phylogeny approach (GBDP) was chosen to rapidly infer trees with branch support values from pairwise whole-genome or single-gene distances.

Gene prediction was performed on the genome assembly by glimmer3^4^ with Hidden Markov models. tRNA, rRNA, and sRNAs recognition made use of tRNAscan-SE (Lowe and Eddy, 1997), RNAmmer, and the Rfam database. The tandem repeats annotation was obtained using the Tandem Repeat Finder,^5^ and the minisatellite DNA and microsatellite DNA were selected based on the number and length of repeat units. The Rapid Annotation using Subsystem Technology (RAST) server was also used to annotate the assembled genomic sequences (Aziz et al., 2008). Functional and pathway analyses were then performed using the BlastKOALA web tool of the KEGG database (Kanehisa et al., 2016). The Resistance Gene Identifier (RGI) platform (version 2) as part of the comprehensive antibiotic resistance database (CARD) was used to automatically annotate DNA sequences based on the CARD, thereby providing antibiotic resistance gene predictions (McArthur et al., 2013).

### Pan-genome analysis

The Bacterial Pan-genome Analysis (BPGA) pipeline was applied for analysis of the genomic diversity of the *Stenotrophomonas* members. The assembled genomic sequences of these strains were predicted by glimmer 3.02, and the protein sequence of other type strains of the genus *Stenotrophomonas* were annotated by RAST 2.0. Pan-genome analysis was performed by BPGA 1.3 using default settings (Chaudhari et al., 2016). A total of 19 protein sequence files annotated from the 19 corresponding strains’ whole genome sequences were used to generate orthologous gene/protein clusters (homologous families) using the USEARCH clustering tool.

### Phenotypic characteristics examination

The strains’ growth was tested on nutrient agar (NA), tryptone soy agar (TSA; Difco), Reasoner’s 2A agar (R2A; Difco), Luria–Bertani agar (LB; Difco), and yeast extract sucrose agar (YM; Difco) at 28°C for 48–72 h. Strains were cultivated at temperatures of 4, 15, 20, 22, 25, 28, 30, 32, 35, 37, 45, and 50°C using TSA media to evaluate growth temperatures. The ability of the three strains to grow at different pH (pH 4–11, at intervals of 1 pH unit) was evaluated using TSB medium, with incubation at 28°C, and monitoring of NaCl tolerance for growth in TSB medium using different final NaCl concentrations (0–10%, w/v, in intervals of 1%; the NaCl contained in TSB medium was also included in calculation of the NaCl concentration), with incubation at 28°C. Antibiotic susceptibility tests were performed using the disk-diffusion plate method on TSA plates using the following antibiotics (37): ampicillin (10 μg), cefaclor (30 μg), chloramphenicol (30 μg), clindamycin (2 μg), erythromycin (15 μg), gentamycin (10 μg), kanamycin (30 μg), netilmicin (30 μg), novobiocin (5 μg), penicillin (10 IU), polymyxin B (300 IU), rifampin (5 μg), vancomycin (30 μg), tetracycline (30 μg), tobramycin (10 μg), and treptomycin (10 μg). Oxidase activity was investigated using the API oxidase reagent (bioMérieux) according to the manufacturer’s instructions. Catalase activity was also evaluated based on the production of bubbles with addition of a drop of 3% (v/v) hydrogen peroxide. Metabolic characteristics were subsequently examined using Biolog GEN III (MicroPlate), API 50CH, and API ZYM test kits (bioMérieux), according to the manufacturer’s instructions. Metabolic results were evaluated after incubation at 28°C for 48–72 h. Other physiological tests, including the ability to produce H_2_S and nitrate reduction, were conducted, as previously described (Yuan et al., 2008). Cellular motility was monitored using the hanging drop method (Jain et al., 2020), and cellular morphological features were observed using a light microscope (Axio A1 Vario, Zeiss) and a transmission electron microscope (JEM-1010, jeol). IAA production was evaluated using the Salkowski colorimetric technique (Ehmann, 1977). Briefly, cultures were incubated for 96 h using the aforementioned culture conditions, then 2 ml of culture broth was centrifuged, followed by treating 1 ml of the culture supernatant with 2 ml of Salkowski reagent (1 ml of 0.5 M FeCl_3_ and 50 ml of 35% HClO_4_; Chaiharn and Lumyong, 2011). Samples were then incubated at 28°C for 30 min in the dark, followed by measuring optical density (OD) values at 530 nm using a spectrophotometer. LB medium containing 10 mg mL^−1^ of tryptophan was used as the blank control. The experiment was conducted in triplicate. The IAA concentration was then calculated according to an IAA standard curve determined with different concentrations of IAA solutions. Chrome azurol sulfonate (CAS) agar plate method (Schwyn and Neilands, 1987) was applied for assessment of siderophore production by these strains. Briefly, 0.05 ml bacterial suspension collected from TSB medium with the test strain growing at 28°C for 48 h, containing of 9 × 10^8^ cells ml^−1^ was spotted over CAS-agar plates in triplicate. After incubation at 28°C for 48 h, recorded the results. On CAS blue agar, if an orange halo formed around colonies, the strain could be judged as siderophore-producer. The ratio of the size of the halo diameter to the colony diameter was larger meant the greater ability of the strain to produce siderophore.

### Chemotaxonomic characterization

Chemotaxonomic characterization was conducted using cells collected after TSB medium cultivation in shake flasks on a rotary shaker (150 r.p.m.) at 28°C for 48 h to achieve logarithmic phase growth. Cellular polar lipids were then extracted, detected using two-dimensional TLC, and identified using previously described procedures (Minnikin et al., 1984). Ubiquinones were extracted and purified according to methods described by Minnikin et al. (1984), followed by analysis with an HPLC instrument (Collins et al., 1977). Cellular fatty acids were extracted, methylated, and analyzed using the Sherlock Microbial Identification System (MIDI) according to the manufacturer’s instructions (Kroppenstedt, 1985). The MIDI Sherlock software program (version 6.0) and the TSBA 6 database were used for the analysis.

### Identification of bacterial isolates using matrix-assisted laser desorption/ionization time-of-flight mass spectrometry

Protein profiles of the newly identified isolates and reference strains were analyzed using matrix-assisted laser desorption/ionization time-of-flight mass spectrometry (MALDI-TOF) mass spectrometry. Cells for proteomic analysis were cultivated at 28°C for 48 h on TSA plates and processed using the ethanol/formic acid method (Bruker Daltonik GmbH). Approximately six clones for each strain were transferred and suspended in 300 μl sterile water, mixed with 900 μl of ethanol, and then centrifuged at 12,000 r.p.m. for 2 min at room temperature. The cell pellet was air-dried and then resuspended in 20 μl of 70% formic acid (Lysis Solution 1, Autobio Diagnostics, China). Then, 20 μl of acetonitrile (Lysis Solution 2, Autobio Diagnostics, China) was added and thoroughly mixed with the solution using a vortex. The mixture was then centrifuged again at 12,000 r.p.m. for 2 min to collect the supernatant. Then, 1 μl of the supernatant was spotted onto the main spectra profile (MSP) 96 target polished steel plate. Air-dried samples were then overlaid with a 1 μl matrix solution (α-cyano-4-hydroxycinnamic acid; Autobio Diagnostics, China) and then air-dried again on the target plate. The target plate was placed in an Autof ms1000 instrument and each site was scanned three times. Thus, each sample comprised eight sites on the target plate and 24 total mass spectra were collected. Spectral data for reference strains were collected using the Autof Acquirer software version 2.0.59 (Autobio Diagnostics, China) and the Autof Analyzer software version 2.0.14 (Autobio Diagnostics, China) to create a local database of main spectra projects (MSPs). Each spectrum was generated using 240 laser shots (six series of 40 laser shots) and with linear positive mode over a mass range of m/z 2,000–20,000. The Autof ms1000 mass was calibrated prior to analysis using a calibrating agent (containing *Escherichia coli* DH5α, ribonuclease, and myoglobin; Autobio Diagnostics, China) as described by He et al. (2022). The spectral data yielded from the test strains were then compared to the database to obtain a score. Scores exceeding 9.5 were defined as highly probable identifications at the species level. Scores between 9.0 and 9.5 indicated highly probable identification at the genus level and probable identification at the species level.

## Results and discussion

### Morphological and physiologic characteristics

The three isolates obtained in this study were identified as Gram-stain negative and aerobic. Strains CPCC 101365^T^, CPCC 101269^T^, and CPCC 101426 grew well on NA and TSA agar, with weak growth on R2A agar. Cells were rod-shaped and motile with a single polar flagellum (Supplementary Figure S1). Growth of all three strains occurred over 22–35°C, with optima of 28–32°C for strain CPCC 101365^T^ and CPCC 101269^T^, except 28–30°C for strain CPCC 101426. Both strains CPCC 101365^T^ and CPCC 101269^T^ grew over the pH range 6.0–8.0, with the optimum of pH 7.0, while strain CPCC 101426 grew over the pH range 6.0–7.0, with an optimum of pH 7.0. The highest NaCl tolerance was 5% (w/v) for strain CPCC 101365^T^ and CPCC 101269^T^, but only 3% (w/v) for strain CPCC 101426. Strains CPCC 101365^T^ and CPCC 101426 also grew well with no more than 2% (w/v) NaCl, while strain CPCC 101269^T^ could grow well with the presence of 3% (w/v) NaCl. All strains exhibited positive catalase and oxidase reactions, but negative tests for starch hydrolysis, cellulose hydrolysis, and H_2_S production. All strains exhibited positive enzymatic activity for acid phosphatase, alkaline phosphatase, esterase (C4), esterase lipase (C8), and naphthol-AS-BI-phosphohydrolase, based on API ZYM strip tests. In addition, all strains exhibited negative tests for α-galactosidase, β-galactosidase, β-glucuronidase, α-mannosidase, and α-fucosidase activity. L-alanine, L-serine, and methyl pyruvate were used by all strains. The strains were all sensitive to chloramphenicol (30 μg), gentamycin (10 μg), kanamycin (30 μg), netilmicin (30 μg), polymyxinB (300 IU), rifampin (5 μg), and tobramycin (10 μg), but resistant to novobiocin (5 μg). In addition, all strains could produce IAA, with the highest IAA concentration (1.8 mg L^−1^) produced by strain CPCC 101426. No siderophore production was detected from any of these three strains. The major differentiating features among strains CPCC 101365^T^, CPCC 101269^T^, CPCC 101426, and other related species are shown in Table 1.

**Table 1:** Physiological characteristics of strains CPCC 101365^T^, CPCC 101269^T^, CPCC 101426, and the closely related *Stenotrophomonas* type strains.

**Characteristic**	**1**	**2**	**3**	**4**	**5**	**6**	**7**	**8**	**9**
pH range with growth	6.0–8.0	6.0–8.0	5.0–9.0	6.0–7.0	6.0–8.0	6.5–8.5	5.0–8.0	6.0–8.0	6.0–7.0
NaCl tolerance (%, w/v)	0–5.0	0–3.0	0–4.0	0–4.0	0–5.0	0–2.5	0–5.0	0–3.0	0–2.0
Hydrolysis of gelatin	−	−	−	+	+	+	+	+	+
Nitrate reduced to nitrite	−	−	−	−	−	+	−	+	−
**Carbon sources used for growth**									
Dextrin	w	−	+	+	+	+	+	−	−
D-maltose	−	−	+	+	+	+	+	−	−
D-trehalose	−	−	−	−	+	+	−	−	−
D-cellobiose	−	−	−	−	+	+	+	−	−
Gentiobiose	w	−	−	−	+	+	+	−	−
Sucrose	−	−	−	−	w	+	w	−	−
D-turanose	−	−	+	−	w	−	w	−	−
*α*-D-lactose	−	−	−	−	+	+	−	−	−
D-melibiose	−	−	−	−	+	+	w	−	−
*β*-methyl-D-glucoside	−	−	−	−	w	+	+	−	−
D-salicin	−	−	−	−	+	+	+	−	−
N-acetyl-D-glucosamine	+	+	w	+	+	+	+	−	−
N-acetyl-β-D-mannosamine	w	−	w	−	+	−	+	−	−
N-acetyl-D-galactosamine	−	+	w	−	w	+	+	−	−
*α*-D-glucose	−	+	−	+	+	+	+	−	−
D-mannose	+	−	+	+	+	+	+	−	−
D-fructose	w	+	+	+	+	−	+	−	−
D-galactose	−	−	−	−	+	−	+	−	−
D-fucose	−	−	−	w	+	−	−	−	−
L-fucose	w	−	w	w	w	−	+	−	−
Inosine	−	−	+	−	−	−	+	−	+
D-glucose-6-PO_4_	−	−	w	−	w	−	+	−	−
D-fructose-6-PO_4_	w	+	+	+	−	−	−	+	w
Gelatin	+	+	+	+	+	+	+	−	+
Glycyl-L-proline	+	+	+	+	+	+	+	−	−
L-arginine	−	−	w	−	−	−	w	+	−
L-aspartic acid	+	−	+	−	+	−	+	−	−
L-glutamic acid	+	+	+	+	+	w	w	+	+
L-histidine	+	+	+	+	w	−	+	−	−
L-serine	+	+	+	+	+	+	+	+	+
Pectin	−	−	w	−	+	−	−	−	−
D-galacturonic acid	w	−	w	w	w	−	−	−	+
L-galactonic acid lactone	w	−	w	−	+	−	−	−	−
D-gluconic acid	−	−	+	−	−	−	−	−	−
Glucuronamide	−	−	−	w	−	−	−	+	w
D-lactic acid methyl ester	−	−	+	−	−	−	w	−	−
L-lactic acid	−	+	+	−	+	−	w	−	−
Citric acid	−	−	+	−	+	+	+	−	+
*α*-keto-glutaric acid	−	−	−	−	+	+	+	−	−
L-malic acid	−	−	−	−	+	+	+	−	−
Bromo-succinic acid	−	−	−	−	+	+	+	−	−
*α*-hydroxy-butyric acid	−	−	+	−	−	−	−	−	−
*β*-hydroxy-D, L-butyric acid	+	+	+	+	−	−	−	−	+
*α*-keto-butyric acid	w	−	+	−	w	−	−	+	+
Acetoacetic acid	+	+	+	−	+	−	+	+	+
Propionic acid	+	+	+	+	+	+	+	−	+
Formic acid	−	−	−	−	−	−	+	−	−
**API ZYM results**									
Esterase lipase (C8)	+	+	+	+	+	+	−	+	−
Lipase (C14)	−	−	−	−	−	−	+	−	w
Leucine arylamidase	+	−	+	+	w	+	+	+	−
Valine arylamidase	+	+	+	+	−	+	−	−	−
Cystine arylamidase	+	−	+	+	−	−	−	−	−
*α*-chymotrypsin	w	−	+	−	−	−	+	−	−
*α*-glucosidase	−	−	+	+	−	−	+	−	−
*β*-glucosidase	−	−	−	−	+	+	−	−	−
N-acetyl-β-glucosamimidase	−	+	+	+	−	−	−	−	−
**Resistance to antibiotics**	clindamycin (2 μg), erythromycin (15 μg), novobiocin (5 μg), penicillin (10 IU), vancomycin (30 μg), tetracycline (30 μg), and treptomycin (10 μg)	ampicillin (10 μg), cefaclor (30 μg), clindamycin (2 μg), novobiocin (5 μg), penicillin (10 IU), and vancomycin (30 μg)	cefaclor (30 μg), clindamycin (2 μg), erythromycin (15 μg), novobiocin (5 μg), and penicillin (10 IU)	ampicillin (10 μg), cefaclor (30 μg), clindamycin (2 μg), neomycin (5 μg), penicillin (10 IU), streptomycin (10 μg), and vancomycin (30 μg)	ampicillin (10 μg), cefaclor (30 μg), clindamycin (2 μg), novobiocin (5 μg), penicillin (10 IU), vancomycin (30 μg), and treptomycin (10 μg)	cefaclor (30 μg), clindamycin (2 μg), novobiocin (5 μg), penicillin (10 IU), and vancomycin (30 μg)	ampicillin (10 μg), cefaclor (30 μg), clindamycin (2 μg), neomycin (5 μg), penicillin (10 IU), and vancomycin (30 μg)	neomycin (5 μg)	neomycin (5 μg), streptomycin (10 μg)

Strains: 1, CPCC 101365^T^; 2, *Stenotrophomonas nitritireducens* JCM 13311^T^; 3. *Stenotrophomonas acidaminiphila* DSM 13117^T^; 4, *Stenotrophomonas daejeonensis* JCM 16244^T^; 5, CPCC 101269^T^; 6, *Stenotrophomonas chelatiphaga* DSM 21508^T^; 7, *Stenotrophomonas tumulicola* JCM 30961^T^; 8, CPCC 101426; 9, *Stenotrophomonas koreensis* JCM 13256^T^.

+, positive; w, weakly positive; −, negative activities/growth.

### Chemotaxonomic properties

Diphosphatidylglycerol (DPG), phosphatidylglycerol (PG), and phosphatidylethanolamine (PE; Supplementary Figure S2) were identified within the polar lipids of strains CPCC 101365^T^, CPCC 101269^T^, and CPCC 101426. The primary respiratory quinone was Q-8. The major fatty acid of strains CPCC 101365^T^ and CPCC 101426 was iso-C_15: 0_. The major fatty acids of strain CPCC 101269^T^ were iso-C_15: 0_, antesio-C_15: 0_, and C_16: 0_ (Supplementary Table S1).

### Phylogenetic analysis

**Figure 1:**
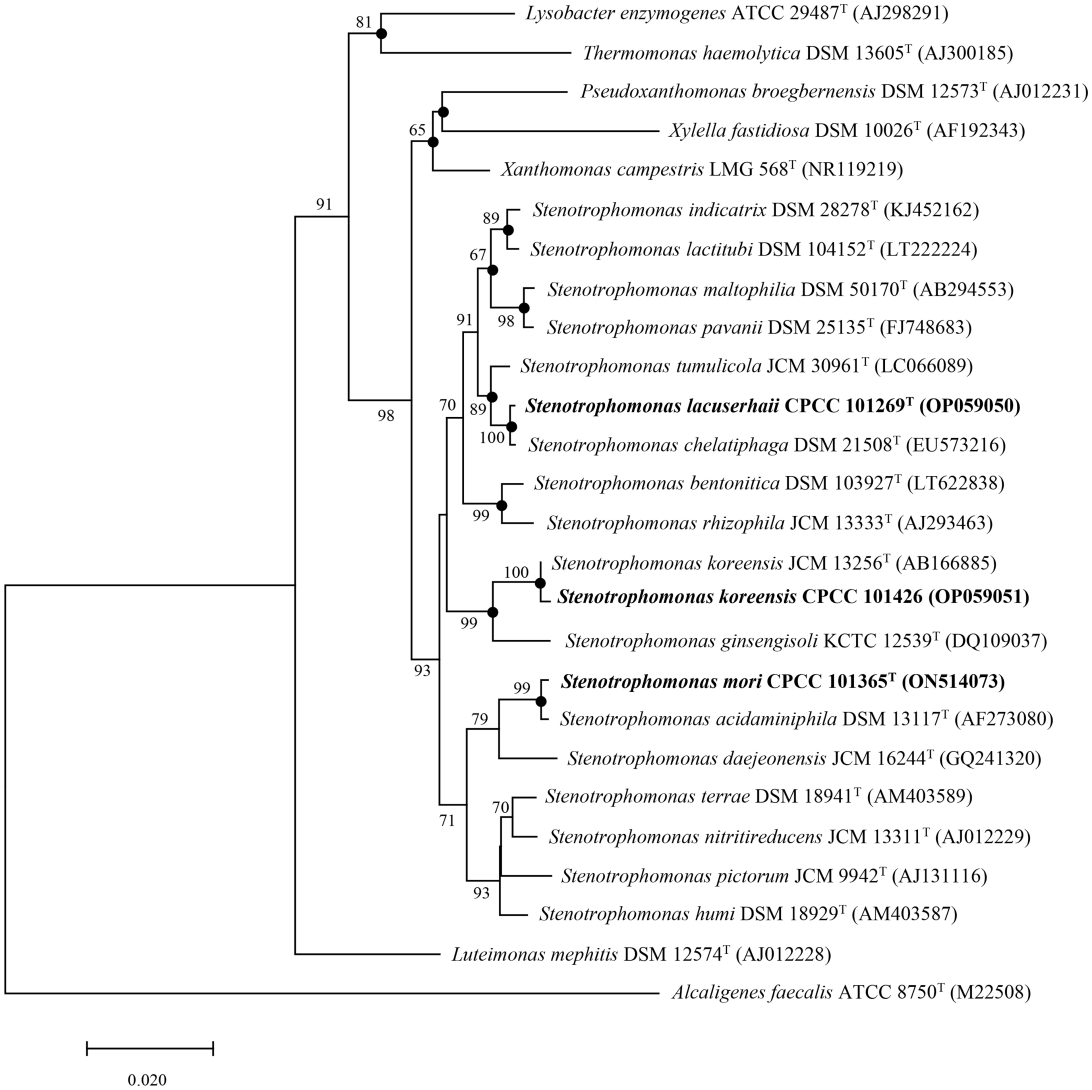
Neighbor-joining tree based on 16S rRNA gene sequences showing the relationships of strains CPCC 101269^T^, CPCC 101365^T^, and CPCC 101426 with other representatives of the family *Lysobacteraceae*. Filled circles indicate that the corresponding nodes were also recovered in phylogenetic trees generated using Maximum Likelihood and Maximum Parsimony methods. Bootstrap values above 50% are shown as percentages of 1,000 replicates. *Alcaligenes faecalis* ATCC 8750^T^ (GenBank accession M22508) was used as the outgroup. Scale bar indicates 0.02 nt substitutions per alignment site.

Nearly complete 16S rRNA gene sequences for strain CPCC 101365^T^ (1,533 bp, accession number ON514073), CPCC 101269^T^ (1,535 bp, accession number OP059050), and CPCC 101426 (1,499 bp, accession number OP059051) were obtained. BLAST searches of the 16S rRNA gene sequences against the GenBank database indicated that strains CPCC 101365^T^, CPCC 101269^T^, and CPCC 101426 exhibited highest similarities with *Stenotrophomonas* species of the family *Lysobacteraceae*. Specifically, the 16S rRNA gene of CPCC 101365^T^ exhibited 99.1% nucleotide similarity to *Stenotrophomonas nitritireducens* JCM 13311^T^ and 96.5–98.8% similarity to 16S rRNA genes from other type strains from various *Stenotrophomonas*. The 16S rRNA gene sequence of strain CPCC 101269^T^ exhibited a nucleotide similarity of 99.9% to that of *Stenotrophomonas chelatiphaga* DSM 21508^T^ and similarities of 97.6–99.4% to those of other type strains from various *Stenotrophomonas* (Supplementary Table S2). Lastly, the 16S rRNA gene sequence of CPCC 101426 exhibited 99.8% nucleotide similarity to that of *S*. *koreensis* JCM 13256^T^ and similarities of 96.9–99.3% to those of other *Stenotrophomonas* members (Supplementary Table S2). Phylogenetic analysis based on 16S rRNA gene sequences confirmed that the strains belonged to the genus *Stenotrophomonas*. The 16S rRNA gene sequence of strain CPCC 101365^T^ formed a stable sub-clade with the type strain *S*. *acidaminiphila* DSM 13117^T^, that were together related to *S*. *daejeonensis* JCM 16244^T^, while that of strain CPCC 101269^T^ formed a robust sub-clade with the 16S rRNA gene of *S*. *chelatiphaga* DSM 21508^T^, that were together associated with *S*. *tumulicola* JCM 30961^T^. Lastly, the 16S rRNA gene sequence of strain CPCC 101426 formed a stable sub-clade with those from *S*. *koreensis* (Figure 1).

In the whole-genome phylogenomic analysis, the genome of strain CPCC 101365^T^ formed a cluster with those of *S*. *nitritireducens* JCM 13311^T^ and *S*. *acidaminiphila* DSM 13117^T^ (Supplementary Figure S3), which slightly contrasted with the 16S rRNA gene-based phylogenetic tree, although the genome comparisons were concordant with comparisons of the 16S rRNA gene similarities. In addition, the phylogenomic analysis presented consistent clustering for strains CPCC 101269^T^ and CPCC 101426, as observed in the 16S rRNA gene-based phylogenetic analysis (Figure 1; Supplementary Figure S3).

### MALDI-TOF MS characterization

The MALDI spectra of strains CPCC 101365^T^ and CPCC 101269^T^, with their phylogenetically related type strains of *Stenotrophomonas,* were differed among each other (Supplementary Figure S4A). The identification score of strain CPCC 101365^T^ was 9.1 when compared with the reference MALDI MS profile of *S*. *nitritireducens* JCM 13311^T^ in the local database, and 8.6 when compared against *S*. *acidaminiphila* DSM 13117^T^ in the database, suggesting that strain CPCC 101365^T^ represents a different species relative to its most closely related species. In particular, some unique peptide peaks in the spectra of strain CPCC 101365^T^ were identified at 6,429.9 and 11,254.6 Da that were not detected in the spectra for *S*. *nitritireducens* JCM 13311^T^ or *S*. *acidaminiphila* DSM 13117^T^ (Supplementary Figure S4B). The identification score of the MALDI MS profile of strain CPCC 101269^T^ was 9.5 when compared against that of *S*. *chelatiphaga* DSM 21508^T^ within the database, and 8.2 when compared with that of *S*. *tumulicola* JCM 30961^T^ in the database, suggesting that strain CPCC 101269^T^ represents a novel species of the genus *Stenotrophomonas*. In addition, a unique peptide peak (at 9,812.5 Da) was observed in the spectra for strain CPCC 101269^T^ that was not detected in the spectra for *S*. *chelatiphaga* DSM 21508^T^ or *S*. *tumulicola* JCM 30961^T^ (Supplementary Figure S4C). The MALDI-TOF MS profile of strain CPCC 101426 exhibited an identification score of 9.7 when compared with the reference MALDI MS profile of *S*. *koreensis* JCM 13256^T^ in the local database, suggesting that strain CPCC 101426 was affiliated with the characterized species *S*. *koreensis*. The majority of peptide peaks within the spectra identified for strain CPCC 101426 essentially coincided with those of *S*. *koreensis* JCM 13256^T^ (± 2 Da; Supplementary Figure S4A).

### Genomic characteristics

The general genomic features of the newly sequenced genomes of these strains and their closely related *Stenotrophomonas* type strains were summarized in Supplementary Table S3. According to the result of CheckM, the completeness of the genomes of strains CPCC 101365^T^, CPCC 101269^T^, and CPCC 101426 was 99.83, 99.59, and 99.07%, and the contamination was 0.34, 1.30, and 0.34%, respectively. The completeness of these genomes was >90, and < 5% contaminated, without being contaminated by the ContEst16S algorithm. The draft genome sizes of strains CPCC 101365^T^, CPCC 101269^T^, and CPCC 101426 were 3.4, 4.0, and 3.1 Mbp, respectively, and were assembled from 13 validated contigs with an N50 length of 468,441 bp; 28 validated contigs with an N50 length of 305,756 bp; and 16 validated contigs with an N50 length of 521,188 bp, respectively. The genomic sequence for strain CPCC 101365^T^ contained 2,969 coding genes, 57 tRNA genes, five rRNA genes, four other ncRNA genes, and 43 pseudogenes. The genome of strain CPCC 101269^T^ contained 3,590 coding genes, 62 tRNA genes, five rRNA genes, four other ncRNA genes, and 53 pseudogenes. The genome of strain CPCC 101426 contained 2,858 coding genes, 76 tRNA genes, six rRNA genes, four other ncRNA genes, and 26 pseudogenes. The genomic G + C content of all three strains ranged between 66.2 and 70.2%. ANI values calculated between strain CPCC 101365^T^, CPCC 101269^T^, and other *Stenotrophomonas* species were all less than 91.8%, and the corresponding dDDH values were below 45.4%. These values were lower than the thresholds used to delineate bacterial species (i.e., ANI < 95% and dDDH <70%; Kim et al., 2014; Supplementary Tables S4; S5). Strain CPCC 101426 and *S*. *koreensis* JCM13256^T^ shared a high ANI value of 97.7% and a high dDDH value of 78.3%, which were consistent with the high level of 16S rRNA gene sequence similarity (99.8%) between the two strains, consistently indicating the assignment of this strain to the species *S*. *koreensis* (Kim et al., 2014).

### Putative functional genes

The phytohormone IAA is responsible for plant-growth promotion and is partially induced/produced by plant-associated bacteria. Gene annotations indicated that the *Stenotrophomonas* strains of this study possessed the potential to produce IAA. All genes necessary for the synthesis of tryptophan, the starting compound for IAA synthesis, were annotated in the genomes of strains CPCC 101365^T^, CPCC 101269^T^, and CPCC 101426 (Supplementary Table S6). Tryptophan biosynthesis usually begins with chorismate that is transformed to anthranilate by anthranilate synthase (TrpEG). Anthranilate is then converted to indole-3-glycerolphosphate in three steps by anthranilate phosphoribosyl transferase (TrpD), phosphoribosylanthranilate isomerase (TrpF), and indole3-glycerol phosphate synthase (TrpC). This compound can be transformed by tryptophan synthase (TrpAB) directly to tryptophan or otherwise enable the generation of indole as an intermediate (Ulrich et al., 2021). In addition, tryptophan can be converted to indole-3-pyruvate (IPA) that can be used in IAA synthesis, while IPA can be directly converted to IAA by indole-pyruvate ferredoxin oxidoreductase (EC 1.2.7.8; Imada et al., 2017; Tullio et al., 2019) that was also encoded by the genomes of strains CPCC 101365^T^ and CPCC 101269^T^.

Partial genes related to coding for the production of siderophores were detected in the genomes of all three strains, including *aroA*, *aroC*, *aroQ*, *pheA*, *tonB*, *exbB*, *exbD*, *tolA*, *tolQ*, *feoB*, *ahpC*, *fiu*, *bfr*, and *fecR* (Supplementary Table S6). It was reported that ferrisiderophores are recognized and taken up *via* TonB-dependent outer membrane receptors in Gram-negative bacteria, which serve as gated porin channels (Koebnik et al., 2000; Ratledge and Dover, 2000; Koebnik, 2005). Binding with the ferrisiderophores, transport is mediated by a complex of inner membrane-anchored proteins *TonB*, *ExbB*, and *ExbD* (Wiener, 2005). And the putative TonB family protein genes, such as *tonB*, *exbB*, and *exbD* were all detected in the genomes of these three strains. While the key enzymes encoding genes *fecBCDE* (Staudenmaier et al., 1989) and *fepBCDG* (Shea and McIntosh, 1991) were not detected in the three genomes, except that only the protein FecR coding gene *fec*R was retrieved from the genomes of the strains CPCC 101365^T^ and CPCC 101426. We analyzed the metabolic pathways for the production of siderophores, and found that the pathways were incomplete, which supported that the negative siderophore production result from the phenotypic experiments.

### Genes associated with antimicrobial resistance

*Stenotrophomonas maltophilia* is an opportunist pathogen with intrinsic resistance to most antibiotics and also exhibits a high capacity to adapt to different environments. Trimethoprim–sulfamethoxazole, β-lactams, carbapenems, fluoroquinolones, tetracyclines, chloramphenicol, aminoglycosides, polymyxins, and macrolides are the most commonly chosen antibiotics for treating infections caused by *S*. *maltophilia* and resistance to these antibiotics is associated with different molecular mechanisms, like multidrug efflux pumps (e.g., the RND, ABC, and MFS families), Qnr (Smqnr), mutations in bacterial topoisomerase and gyrase encoding genes, lipopolysaccharides (SpgM), and intrinsic and acquired β-lactamases (Brooke, 2021). To investigate whether the three strains exhibited similar drug resistance characteristics as *S*. *maltophilia*, antibiotic resistance genes in the genomes of these strains were investigated. The antibiotic resistance gene annotations for strains CPCC 101365^T^, CPCC 101269^T^, CPCC 101426, *S*. *maltophilia* DSM 50170^T^, and the human pathogen *S*. *maltophilia* K279a are shown in Supplementary Figure S5. The number of antibiotic efflux and β-lactamase homolog genes exhibited the most significant differences among these genomes. The number of related homolog genes in the genomes of strain *S*. *maltophilia* DSM 50170^T^ (284 antibiotic efflux homologs and 31 β-lactamase homologs) and *S*. *maltophilia* K279a (288 antibiotic efflux homologs and 29 β-lactamase homologs) was much higher than for the genomes of strain CPCC 101365^T^ (189 and 19 homologs, respectively), CPCC 101269^T^ (283 and 14 homologs, respectively), and CPCC 101426 (133 and 14 homologs, respectively). Comparison against the CARD database revealed that *adeF* efflux pump genes were present in all of the above genomes. The gene product of *adeF* is a fluoroquinolone and tetracycline antibiotic efflux pump. However, some genes were specific to the genomes of *S*. *maltophilia* K279a and *S*. *maltophilia* DSM 50170^T^ that coded for antibiotic resistance and multidrug efflux pumps that were absent from the genomes of strains CPCC 101365^T^, CPCC 101269^T^, and CPCC 101426. For example, *smeR* was annotated as a resistance-nodulation-cell division (RND) antibiotic efflux pump gene that was only present in the genomes of *S*. *maltophilia* DSM 50170^T^ and *S*. *maltophilia* K279a, which was implicated in resistance to beta-lactams, aminoglycosides, and quinolones. However, *smeR* was not present in the genomes of strain CPCC 101365^T^, CPCC 101269^T^, and CPCC 101426.

### Pan-genome features

**Figure 2:**
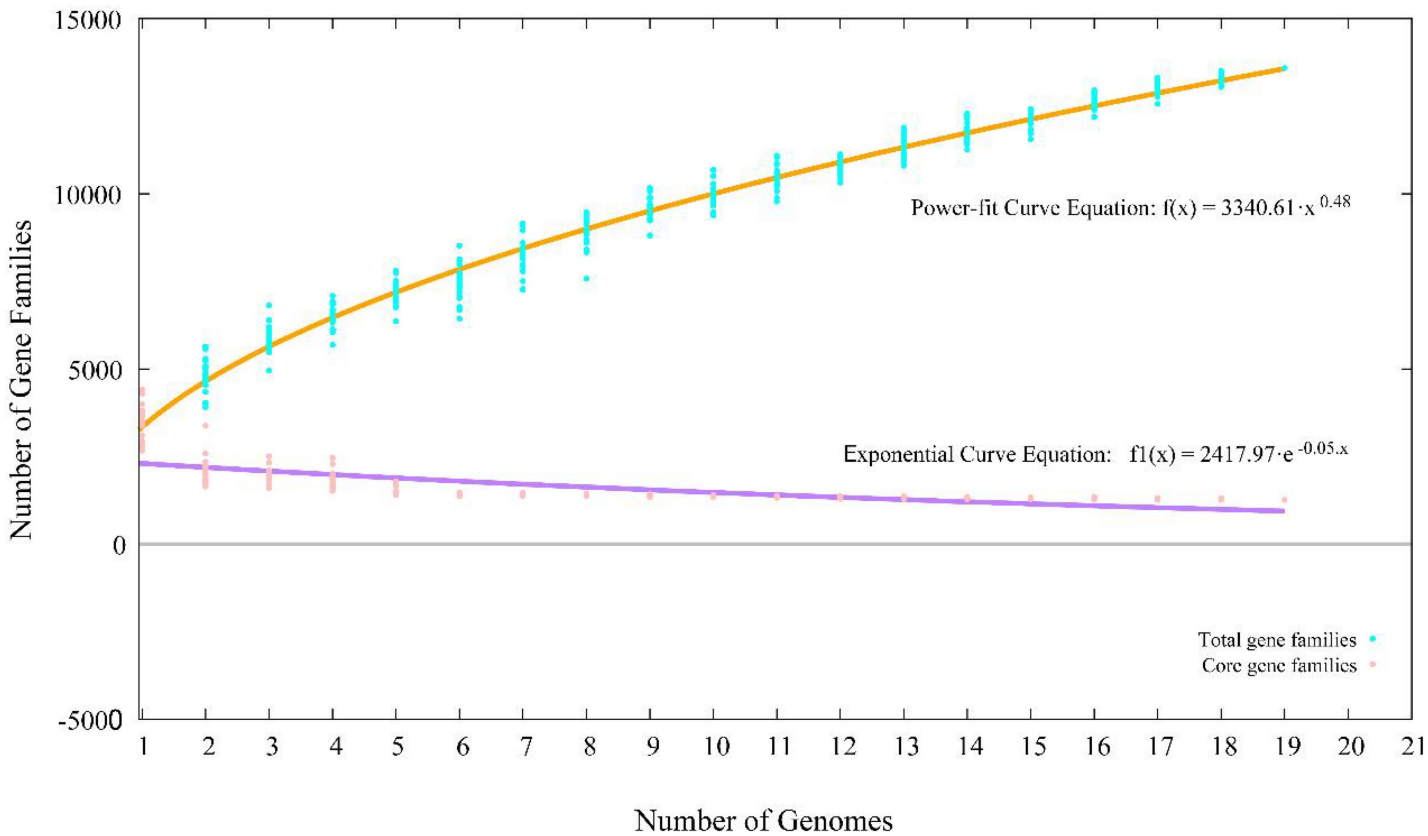
Pan-core plot of the genomes of CPCC 101365^T^, CPCC 101269^T^, CPCC 101426, and other 16 type strains of the genus *Stenotrophomonas*. *x* axis shows the number of genomes while the *y* axis represents the number of gene families.

A total of 65,534 protein-coding genes (Table 2) were sorted from the genomes of these 19 strains of the genus *Stenotrophomonas*, which were divided into 13,592 homologous families by cluster analysis. Among them, there were a total of 1,258 core genes commonly shared by these 19 strains, which were defined as core genome here, accounting for about 36.5% of the pan-genome of the 19 strains of the genus *Stenotrophomonas*. The proportion of the unique genes (6,101 genes) was about 44.9%. Core and pangenome analyses of the 19 genomes the genus *Stenotrophomonas* revealed that the “open” pangenome fitted into a powerlaw regression function [*f*(X) = 3340.61X^0.48^], while the core genome was fitted into an exponential regression [*f*(X) = 2417.97e^−0.05X^; Figure 2]. The open pangenome suggested that the genus *Stenotrophomonas* has undergone considerable gene exchanging to extend their functional profiles such as horizontal gene transfer (HGT; Thomas and Nielsen, 2005). Out of 13,592 genes/clusters, BPGA could map 3,732 genes (27.5%) to Kyoto Encyclopedia of Genes and Genomes (KEGG) pathways, including core genes (1,326, 35.5%), accessory genes (1,719, 46.1%), and unique genes (687, 18.4%). After filtering the KEGG pathway related to eukaryotes, we obtained an overview on the metabolic pathway (>1%) corresponding to the gene(s) in the pan-genome of the genus *Stenotrophomonas*. Unique genes (687) seemed to be mainly enriched in human diseases related sub-category (17.2%), signal transduction (11.9%), carbohydrate metabolism (11.4%), some other elementary metabolism (7.0%), amino acid metabolism (6.6%), lipid metabolism (5.4%), xenobiotics biodegradation and metabolism (4.4%), membrane transport (4.2%), organismal systems related sub-category (3.6%), energy metabolism (2.6%), nucleotide metabolism (2.3%), cell motility (2.2%), replication and repair (2.0%), metabolism of cofactors and vitamins (1.9%), glycan biosynthesis and metabolism (1.9%), metabolism of terpenoids and polyketides (1.6%), folding, sorting, and degradation (1.5%), and metabolism of other amino acids (1.0%). Interestingly, tetracycline resistance transcriptional repressor TetR (K5L01_RS13290) coding gene (*tetR*) was predicted as the unique gene in the genome of strain CPCC 101365^T^. This protein regulates transcription of a family of tetracycline resistance determinants in Gram-negative bacteria, which was probably derived from horizontal gene transfer. Some genes related to siderophore uptake and transport were also predicted in the unique genes of the plant endophytic strain CPCC 101269^T^, such as, catecholate siderophore receptor coding gene *fiu*. In the core genome of the strains CPCC 101365^T^, CPCC 101269^T^, and CPCC 101426, some genes related to the biosynthesis of indole-3-acetic acid were retrieved (Supplementary Table S6).

**Table 2:** The pan-genome profile information of the genus *Stenotrophomonas*.

Genome number	Organism name	No. of core genes	No. of accessory genes	No. of unique genes	No. of exclusively absent genes
1	CPCC 101269^T^	1,258	1,917	272	3
2	CPCC 101365^T^	1,258	1,328	246	25
3	CPCC 101426	1,258	1,382	78	3
4	*S*. *acidaminiphila* DSM 13117^T^	1,258	1,776	342	2
5	*S*. *bentonitica* DSM 103927^T^	1,258	2,059	494	8
6	*S*. *chelatiphaga* DSM 21508^T^	1,258	1,868	248	14
7	*S*. *daejeonensis* JCM 16244^T^	1,258	1,346	252	19
8	*S*. *ginsengisoli* KCTC 12539^T^	1,258	1,277	408	22
9	*S*. *humi* DSM 18929^T^	1,258	1,904	349	8
10	*S*. *indicatrix* DSM 28278^T^	1,258	2,523	204	0
11	*S*. *koreensis* JCM 13256^T^	1,258	1,251	158	11
12	*S*. *lactitubi* DSM 104152^T^	1,258	2,635	400	2
13	*S*. *maltophilia* NCTC 10257^T^	1,258	2,604	546	1
14	*S*. *nitritireducens* JCM 13311^T^	1,258	1773	380	50
15	*S*. *pavanii* DSM 25135^T^	1,258	2,353	169	11
16	*S*. *pictorum* JCM 9942^T^	1,258	1,541	303	6
17	*S*. *rhizophila* DSM 14405^T^	1,258	1,970	268	4
18	*S*. *terrae* DSM 18941^T^	1,258	1,952	467	13
19	*S*. *tumulicola* JCM 30961^T^	1,258	1,862	517	8

### Distinguishing *Stenotrophomonas* isolates from the potential human pathogen *Stenotrophomonas maltophilia*

The capacity of bacteria to grow at 37°C is a major criterion used to distinguish pathogenic and non-pathogenic *S*. *maltophilia* and *S*. *rhizophila* strains. *S*. *rhizophila* DSM 14405^T^ cannot survive inside the human body, partly due to the absence of heat shock genes and genes involved in a suicide mechanism that can be up-regulated when temperatures increase (Berg and Martinez, 2015). Alavi et al. (2014) observed that *S*. *rhizophila* was in an advanced state of shock at 35°C, and that the primary gene encoding Sigma factor 24 was strongly expressed during the early phase of cellular stress. The genes encoding RpoH (heat shock-specific Sigma factor 32), specific chaperone (*fkpA*), and protease (*degP*) were down-regulated, while other response mechanisms were upregulated, and the heat shock-specific response mechanism was strongly activated. The upregulation of genes coding for toxin/antidote-based suicide systems further illustrates the adaptive mechanisms of *S*. *rhizophila* DSM 14405^T^ and highlight its response mechanism to severe heat stress. Genes coding for chaperones involved in both general stress and heat shock specific responses including *dnaJ-K*, *groS-L,* and *htpG*, in addition to the gene coding for the heat shock specific sigma factor including *rpoH*, and numerous genes coding for proteases that degrade stress-denatured proteins were all detected in the genomes of strains CPCC 101365^T^, CPCC 101269^T^, CPCC 101426, *S*. *maltophilia* DSM 50170^T^, and the human pathogen *S*. *maltophilia* K279a (Supplementary Table S7). Further, genes related to the toxin/antidote suicide system were also detected in the genomes of these strains. These results were not consistent with the results of *S*. *rhizophila* DSM 14405^T^, so the presence or absence of these genes in genome could not be used to distinguish pathogenic and non-pathogenic strains of the genus *Stenotrophomonas*. In addition, the gene encoding for a biosynthetic enzyme for glucosylglycerol, glucosylglycerolphosphate synthase (*ggpS*), can improve tolerance to salinity and salt stress. *Stenotrophomonas rhizophila* DSM14405^T^ possesses *ggpS*, which are essential for the synthesis and transport of the important osmolyte glucosylglycerol, while *ggpS* is absent in the genomes of the human pathogen *S*. *maltophilia* K279a (Alavi et al., 2014). In this study, the *ggpS* gene was present in the genome of strain CPCC 101269^T^, but was absent from the genomes of strain CPCC 101365^T^ and CPCC 101426. So the presence or absence of *ggpS* gene in genome could not be used to distinguish pathogenic and non-pathogenic strains of the genus *Stenotrophomonas*. Alternatively, we speculated that differences between environmental strains and those causing infections might occur at the level of gene regulation, rather than *via* the presence or absence of genes (Alonso et al., 1999). Thus, criteria for distinguishing non-pathogenic strains of the genus *Stenotrophomonas* from *S*. *maltophilia* that are pathogenic to humans requires further research.

## Conclusion

In this study, three *Stenotrophomonas* strains (CPCC 101365^T^, CPCC 101269^T^, and CPCC 101426) were isolated from a surface-sterilized medicinal plant root of mulberry collected from Chuxiong of the Yunnan Province, fresh water from Erhai Lake in the Yunnan Province, and sandy soil from the Badain Jaran desert in Inner Mongolia Autonomous Region, respectively. Phylogenetic analysis based on 16S rRNA genes and whole genomes confirmed that the isolates should be assigned to the genus *Stenotrophomonas*. The similarities of 16S rRNA gene sequences from these three isolates and other validly identified *Stenotrophomonas* strains were above 98.65%, which has been previously used as the threshold for differentiating species. However, strains CPCC 101365^T^ and CPCC 101269^T^ likely represent two distinct new species within the genus *Stenotrophomonas* based on their low ANI (< 95%) and dDDH (< 70%) values in comparison to each other and all other type species of the genus *Stenotrophomonas* (Kim et al., 2014). Further, combined phenotypic and genotypic data consistently indicated that strains CPCC 101365^T^ and CPCC 101269^T^ differed from other *Stenotrophomonas* species and represented two novel species, while strain CPCC 101426 was affiliated with *Stenotrophomonas koreensis*. Lastly, the genomes of strains CPCC 101365^T^, CPCC 101269^T^, and CPCC 101426 encoded proteins associated with plant growth promotion, and these effects were confirmed with phenotypic tests.

Given the diversity of *Stenotrophomonas* spp. in ecological distribution, gene components, potential ecological function, and undiscovered novel species, we suppose that such microorganisms deserve study in depth. The deep phylo-taxono-genomics investigation on the siblings exemplified how to clarify the relationship between these microorganisms in the omics era (Bansal et al., 2021).

### Description of *Stenotrophomonas mori* sp. nov.

*Stenotrophomonas mori* (mo′ri. L. gen. n. *mori* of a mulberry tree, of *Morus*, the generic name of the white mulberry, *Morus alba* L., from which the type strain was isolated).

Cells are Gram-staining-negative, non-motile, non-spore-forming, aerobic, and rod. Colonies on TSA medium are convex with an entire margin, viscous, and semi-translucent after 48 h at 28°C (pH 7.0). Growth occurs at 22–35°C and pH 6.0–8.0, with optimum at 28–32°C and pH 7.0, respectively. NaCl is not required for growth, but NaCl tolerance is up to 5.0% (w/v). Catalase-positive and oxidase-positive, negative for H_2_S, nitrate reduced to nitrite, starch, cellulose, and hydrolysis of gelatin tests. Positive for acid phosphatase, alkaline phosphatase, cystine arylamidase, esterase lipase (C8), esterase (C4), leucine arylamidase, Naphthol-AS-BI-phosphohydrolase, trypsin, and valine arylamidase. Acetic acid, acetoacetic acid, D-Mannose, gelatin, glycyl-L-proline, L-Aspartic acid, L-Glutamic Acid, L-Histidine, methyl Pyruvate, N-Acetyl-D-Glucosamine, propionic acid, β-Hydroxy-D, and L-butyric acid can be used as the sole carbon source, and acid is produced from D-fructose, D-glucose, D-mannose, esculin ferric citrate, and N-acetylglucosamine. The respiratory quinone is ubiquinone-8. Diphosphatidylglycerol, phosphatidylglycerol, and phosphatidylethanolamine are detected in the polar lipids extraction. The major cellular fatty acid is Iso-C_15: 0_. The type strain CPCC 101365^T^ (= DY006^T^ = KCTC 82900^T^) was isolated from a mulberry parasitic medicinal plant sample from Yunnan, China. The genome of the type strain is characterized by a size of 3.4 Mbp and the genomic G + C content of 70.2%.

### Description of *Stenotrophomonas lacuserhaii* sp. nov.

*Stenotrophomonas lacuserhaii* (la.cus.er.ha’i.i. L. masc. n. *lacus*, lake; N.L. gen. n. *lacuserhaii*, from lake Erhai, the reservoir from which the type strain was obtained).

Cells are Gram-staining-negative, non-motile, non-spore-forming, aerobic, and rod. Colonies on TSA medium are convex with an entire margin, viscous, and semi-translucent after 48 h at 28°C (pH 7.0). Growth occurs at 22–35°C and pH 6.0–8.0, with optimum at 28–32°C and pH 7.0, respectively. NaCl is not required for growth, but NaCl tolerance is up to 5.0% (w/v). Catalase-positive and oxidase-positive. Positive for hydrolysis of gelatin test, negative for H_2_S, nitrate reduced to nitrite, starch, and cellulose tests. Positive for acid phosphatase, alkaline phosphatase, esterase (C4), esterase lipase (C8), naphthol-AS-BI-phosphohydrolase, and *β*-glucosidase. Acetic acid, acetoacetic acid, bromo-succinic acid, citric acid, D-cellobiose, dextrin, D-fructose, D-fucose, D-galactose, D-maltose, D-mannose, D-melibiose, D-salicin, D-trehalose, gelatin, gentiobiose, glycyl-L-proline, L-alanine, L-aspartic acid, L-galactonic acid lactone, L-glutamic acid, L-lactic acid, L-malic acid, L-serine, methyl pyruvate, N-acetyl-D-glucosamine, N-acetyl-β-D-mannosamine, pectin, propionic acid, tween 40, *α*-D-glucose, *α*-D-lactose, and *α*-keto-glutaric acid can be used as the sole carbon source, and acid is produced from D-fructose, D-fucose, D-galactose, D-glucose, D-maltose, D-mannose, esculin ferric citrate, N-acetylglucosamine, and potassium 2-ketogluconate. The respiratory quinone is ubiquinone-8. Diphosphatidylglycerol, phosphatidylglycerol, and phosphatidylethanolamine are detected in the polar lipids extraction. The major cellular fatty acids are iso-C_15: 0_, antesio-C_15: 0_, and C_16: 0_. The type strain CPCC 101269^T^ (=K32^T^ = KCTC 82901^T^) was isolated from a water sample collected from Erhai Lake, China. The genome of the type strain is characterized by a size of 4.0 Mbp and the genomic G + C content of 66.4%.

## Data availability statement

The datasets presented in this study can be found in online repositories. The names of the repository/repositories and accession number(s) can be found in the article/Supplementary material.

## Author contributions

YD, X-FH, and Z-MJ carried out the experiments. YD, Z-MJ, and Y-QZ designed the research, analyzed the data, and prepared the manuscript. L-YY and YL collected the samples. All authors contributed to the article and approved the submitted version.

## Funding

This research was supported by the CAMS Innovation Fund for Medical Sciences (CIFMS, 2021-I2M-1-055), the National Natural Science Foundation of China (32170021), the Beijing Natural Science Foundation (5212018), the key project at Central government level-the ability establishment of sustainable use for valuable Chinese medicine resources (2060302), and the National Infrastructure of Microbial Resources (NIMR-2021-3).

## Conflict of interest

The authors declare that the research was conducted in the absence of any commercial or financial relationships that could be construed as a potential conflict of interest.

## Publisher’s note

All claims expressed in this article are solely those of the authors and do not necessarily represent those of their affiliated organizations, or those of the publisher, the editors and the reviewers. Any product that may be evaluated in this article, or claim that may be made by its manufacturer, is not guaranteed or endorsed by the publisher.

## Footnotes

^1^ https://lpsn.dsmz.de/genus/*Stenotrophomonas*

^2^ http://www.ezbiocloud.net/

^3^ www.ezbiocloud.net/tools/contest16s

^4^ http://www.cbcb.umd.edu/software/glimmer/

^5^ http://tandem.bu.edu/trf/trf.html

## Abbreviations

DPG: diphosphatidylglycerol
PG: phosphatidylglycerol
PE: phosphatidylethanolamine
ANI: average nucleotide identity
dDDH: digital DNA–DNA hybridization
IAA: indole-3-acetic acid
MALDI-TOF: MS, matrix-assisted laser desorption/ionization time-of-flight mass spectrometry

## References

[ref1] Alavi P. Starcher M. R. Thallinger G. G. Zachow C. Muller H. Berg G. (2014). *Stenotrophomonas* comparative genomics reveals genes and functions that differentiate beneficial and pathogenic bacteria. BMC Genomics 15:482. doi: 10.1186/1471-2164-15-482, PMID: 24939220 PMC4101175

[ref2] Alonso A. Rojo F. Martínez J. L. (1999). Environmental and clinical isolates of *Pseudomonas aeruginosa* show pathogenic and biodegradative properties irrespective of their origin. Environ. Microbiol. 1, 421–430. doi: 10.1046/j.1462-2920.1999.00052.x, PMID: 11207762

[ref3] Assih E. A. Ouattara A. S. Thierry S. Cayol J. L. Labat M. Macarie H. (2002). *Stenotrophomonas acidaminiphila* sp. nov., a strictly aerobic bacterium isolated from an upflow anaerobic sludge blanket (UASB) reactor. Int. J. Syst. Evol. Microbiol. 52, 559–568. doi: 10.1099/00207713-52-2-559 11931169

[ref4] Auch A. F. Von Jan M. Klenk H. P. Göker M. (2010). Digital DNA–DNA hybridization for microbial species delineation by means of genome-to-genome sequence comparison. Stand. Genomic Sci. 2, 117–134. doi: 10.4056/sigs.531120, PMID: 21304684 PMC3035253

[ref5] Aziz R. K. Bartels D. Best A. A. Dejongh M. Disz T. Edwards R. A.. (2008). The RAST server: rapid annotations using subsystems technology. BMC Genomics 9:75. doi: 10.1186/1471-2164-9-75, PMID: 18261238 PMC2265698

[ref6] Bansal K. Kumar S. Kaur A. Singh A. Patil P. B. (2021). Deep phylo-taxono genomics reveals Xylella as a variant lineage of plant associated *Xanthomonas* and supports their taxonomic reunification along with *Stenotrophomonas* and Pseudoxanthomonas. Genomics 113, 3989–4003. doi: 10.1016/j.ygeno.2021.09.021, PMID: 34610367

[ref7] Berg G. Martinez J. L. (2015). Friends or foes: can we make a distinction between beneficial and harmful strains of the *Stenotrophomonas maltophilia* complex? Front. Microbiol. 6:241. doi: 10.3389/fmicb.2015.00241, PMID: 25873912 PMC4379930

[ref8] Brooke J. S. (2021). Advances in the microbiology of *Stenotrophomonas maltophilia*. Clin. Microbiol. Rev. 34:e0003019. doi: 10.1128/cmr.00030-19, PMID: 34043457 PMC8262804

[ref9] Chaiharn M. Lumyong S. (2011). Screening and optimization of indole-3-acetic acid production and phosphate solubilization from rhizobacteria aimed at improving plant growth. Curr. Microbiol. 62, 173–181. doi: 10.1007/s00284-010-9674-6, PMID: 20552360

[ref10] Chaudhari N. M. Gupta V. K. Dutta C. (2016). BPGA- an ultra-fast pan-genome analysis pipeline. Sci. Rep. 6:24373. doi: 10.1038/srep24373, PMID: 27071527 PMC4829868

[ref11] Coenye T. Vanlaere E. Falsen E. Vandamme P. Vandamme P. (2004). Stenotrophomonas africana Drancourt et al. 1997 is a later synonym of Stenotrophomonas maltophilia (Hugh 1981) Palleroni and Bradbury 1993. Int. J. Syst. Evol. Microbiol. 54, 1235–1237. doi: 10.1099/ijs.0.63093-0, PMID: 15280297

[ref12] Collins M. D. Pirouz T. Goodfellow M. Minnkin D. E. (1977). Distribution of Menaquinones in Actinomycetes and Corynebacteria. J. Gen. Microbiol. 100, 221–230. doi: 10.1099/00221287-100-2-221, PMID: 894261

[ref13] Dubey K. K. Fulekar M. H. (2012). Chlorpyrifos bioremediation in Pennisetum rhizosphere by a novel potential degrader *Stenotrophomonas maltophilia* MHF ENV20. World J. Microbiol. Biotechnol. 28, 1715–1725. doi: 10.1007/s11274-011-0982-1, PMID: 22805954

[ref14] Ehmann A. (1977). The van urk-Salkowski reagent--a sensitive and specific chromogenic reagent for silica gel thin-layer chromatographic detection and identification of indole derivatives. J. Chromatogr. 132, 267–276. doi: 10.1016/s0021-9673(00)89300-0 188858

[ref15] Felsenstein J. (1981). Evolutionary trees from DNA sequences: a maximum likelihood approach. J. Mol. Evol. 17, 368–376. doi: 10.1007/bf01734359 7288891

[ref16] Felsenstein J. (1985). Confidence limits on phylogenies: an approach using the bootstrap. Evolution 39, 783–791. doi: 10.1111/j.1558-5646, PMID: 28561359

[ref17] Finkmann W. Altendorf K. Stackebrandt E. Lipski A. (2000). Characterization of N_2_O-producing *Xanthomonas*-like isolates from biofilters as *Stenotrophomonas nitritireducens* sp. nov., *Luteimonas mephitis* gen. Nov., sp. nov. and *Pseudoxanthomonas broegbernensis* gen. Nov., sp. nov. Int. J. Syst. Evol. Microbiol. 50, 273–282. doi: 10.1099/00207713-50-1-273, PMID: 10826814

[ref18] Handa Y. Tazato N. Nagatsuka Y. Koide T. Kigawa R. Sano C.. (2016). *Stenotrophomonas tumulicola* sp. nov., a major contaminant of the stone chamber interior in the Takamatsuzuka tumulus. Int. J. Syst. Evol. Microbiol. 66, 1119–1124. doi: 10.1099/ijsem.0.000843, PMID: 26653171

[ref19] He C. Feng J. Su J. Zhang T. Yu L. (2022). Application of matrix-assisted laser desorption/ionization time-of-flight mass spectrometry for the rapid identification of yeast species from polar regions. Front. Microbiol. 13:832893. doi: 10.3389/fmicb.2022.832893, PMID: 35283859 PMC8905632

[ref20] Hugh R. Leifson E. (1953). The taxonomic significance of fermentative versus oxidative metabolism of carbohydrates by various gram negative bacteria. J. Bacteriol. 66, 24–26. doi: 10.1128/jb.66.1.24-26.1953, PMID: 13069461 PMC357086

[ref21] Hugh R. Leifson E. (1963). A description of the type strain of *pseudomonas maltophilia*. Int. J. Syst. Evol. Microbiol. 13, 133–138. doi: 10.1099/0096266X-13-3-133

[ref22] Hugh R. Ryschenkow E. (1961). *Pseudomonas maltophilia*, an *Alcaligenes*-like species. J. Gen. Microbiol. 26, 123–132. doi: 10.1099/00221287-26-1-123, PMID: 14449786

[ref23] Ikemoto S. Suzuki K. Kaneko T. Komagata K. (1980). Characterization of strains of *pseudomonas maltophilia* which do not require methionine. Int. J. Syst. Bacteriol. 30, 437–447. doi: 10.1099/00207713-30-2-437

[ref24] Imada E. L. Rolla Dos Santos A. A. P. Oliveira A. L. M. Hungria M. Rodrigues E. P. (2017). Indole-3-acetic acid production via the indole-3-pyruvate pathway by plant growth promoter *rhizobium tropici* CIAT 899 is strongly inhibited by ammonium. Res. Microbiol. 168, 283–292. doi: 10.1016/j.resmic.2016.10.010, PMID: 27845247

[ref25] Jain A. Jain R. Jain S. (2020). Motility Testing–Hanging Drop Method and Stab. Basic Techniques in Biochemistry, Microbiology and Molecular Biology, pp. 121–122. New York, NY: Humana.

[ref26] Kanehisa M. Sato Y. Kawashima M. Furumichi M. Tanabe M. (2016). KEGG as a reference resource for gene and protein annotation. Nucleic Acids Res. 44, D457–D462. doi: 10.1093/nar/gkv1070, PMID: 26476454 PMC4702792

[ref27] Kaparullina E. Doronina N. Chistyakova T. Trotsenko Y. (2009). *Stenotrophomonas chelatiphaga* sp. nov., a new aerobic EDTA-degrading bacterium. Syst. Appl. Microbiol. 32, 157–162. doi: 10.1016/j.syapm.2008.12.003, PMID: 19216044

[ref28] Kim M. Oh H. S. Park S. C. Chun J. (2014). Towards a taxonomic coherence between average nucleotide identity and 16S rRNA gene sequence similarity for species demarcation of prokaryotes. Int. J. Syst. Evol. Microbiol. 64, 346–351. doi: 10.1099/ijs.0.059774-0, PMID: 24505072

[ref29] Kim H. B. Srinivasan S. Sathiyaraj G. Quan L. H. Kim S. H. Bui T. P. N.. (2010). *Stenotrophomonas ginsengisoli* sp. nov., isolated from a ginseng field. Int. J. Syst. Evol. Microbiol. 60, 1522–1526. doi: 10.1099/ijs.0.014662-0, PMID: 19684314

[ref30] Kimura M. (1979). The neutral theory of molecular evolution. Sci. Am. 241, 98–126. doi: 10.1038/scientificamerican1179-98 504979

[ref31] Kimura M. (1980). A simple method for estimating evolutionary rates of base substitutions through comparative studies of nucleotide sequences. J. Mol. Evol. 16, 111–120. doi: 10.1007/bf01731581, PMID: 7463489

[ref32] Kluge A. G. Farris J. S. (1969). Quantitative phyletics and the evolution of anurans. Syst. Zool. 18, 1–32. doi: 10.2307/2412407

[ref33] Koebnik R. (2005). TonB-dependent trans-envelope signalling: the exception or the rule? Trends Microbiol. 13, 343–347. doi: 10.1016/j.tim.2005.06.005 15993072

[ref34] Koebnik R. Locher K. P. Van Gelder P. (2000). Structure and function of bacterial outer membrane proteins: barrels in a nutshell. Mol. Microbiol. 37, 239–253. doi: 10.1046/j.1365-2958.2000.01983.x, PMID: 10931321

[ref35] Komagata K. Yabuuchi E. Tamagawa Y. Ohyama A. (1974). *Pseudomonas melanogena* Iizuka and Komagata 1963, a later subjective synonym of *pseudomonas maltophilia* Hugh and Ryschenkow 1960. Int. J. Syst. Bacteriol. 24, 242–247. doi: 10.1099/00207713-24-2-242

[ref36] Kroppenstedt R. (1985). Fatty acid and menaquinone analysis of actinomycetes and related organisms. Soc. Appl. Bacteriol. Tech. Ser. 20, 173–199.

[ref37] Kumar S. Stecher G. Li M. Knyaz C. Tamura K. (2018). MEGA X: molecular evolutionary genetics analysis across computing platforms. Mol. Biol. Evol. 35, 1547–1549. doi: 10.1093/molbev/msy096, PMID: 29722887 PMC5967553

[ref38] Lee M. Woo S. G. Chae M. Shin M. C. Jung H. M. Ten L. N. (2011). *Stenotrophomonas daejeonensis* sp. nov., isolated from sewage. Int. J. Syst. Evol. Microbiol. 61, 598–604. doi: 10.1099/ijs.0.017780-0, PMID: 20400671

[ref39] Li W. J. Xu P. Schumann P. Zhang Y. Q. Pukall R. Xu L. H.. (2007). *Georgenia ruanii* sp. nov., a novel actinobacterium isolated from forest soil in Yunnan (China), and emended description of the genus *Georgenia*. Int. J. Syst. Evol. Microbiol. 57, 1424–1428. doi: 10.1099/ijs.0.64749-0 17625169

[ref40] Lowe T. M. Eddy S. R. (1997). tRNAscan-SE: a program for improved detection of transfer RNA genes in genomic sequence. Nucleic Acids Res. 25, 955–964. doi: 10.1093/nar/gkab688, PMID: 9023104 PMC146525

[ref41] Mcarthur A. G. Waglechner N. Nizam F. Yan A. Azad M. A. Baylay A. J.. (2013). The comprehensive antibiotic resistance database. Antimicrob. Agents Chemother. 57, 3348–3357. doi: 10.1128/aac.00419-13, PMID: 23650175 PMC3697360

[ref42] Meier-Kolthoff J. P. Göker M. (2019). TYGS is an automated high-throughput platform for state-of-the-art genome-based taxonomy. Nat. Commun. 10:2182. doi: 10.1038/s41467-019-10210-3, PMID: 31097708 PMC6522516

[ref43] Minnikin D. E. Odonnell A. G. Goodfellow M. Alderson G. Athalye M. Schaal A.. (1984). An integrated procedure for the extraction of bacterial isoprenoid quinones and polar lipids. J. Microbiol. Methods 2, 233–241. doi: 10.1016/0167-7012(84)90018-6

[ref44] Nakayama T. Homma Y. Hashidoko Y. Mizutani J. Tahara S. (1999). Possible role of xanthobaccins produced by *Stenotrophomonas* sp. strain SB-K88 in suppression of sugar beet damping-off disease. Appl. Environ. Microbiol. 65, 4334–4339. doi: 10.1128/AEM.65.10.4334-4339, PMID: 10508056 PMC91574

[ref45] Oves M. Khan M. S. Zaidi A. Ahmed A. S. Ahmed F. Ahmad E.. (2013). Antibacterial and cytotoxic efficacy of extracellular silver nanoparticles biofabricated from chromium reducing novel OS4 strain of *Stenotrophomonas maltophilia*. PLoS One 8:e59140. doi: 10.1371/journal.pone.0059140, PMID: 23555625 PMC3605433

[ref46] Palleroni N. J. Bradbury J. F. (1993). *Stenotrophomonas*, a new bacterial genus for *Xanthomonas maltophilia* (Hugh 1980) Swings et al. 1983. Int. J. Syst. Bacteriol. 43, 606–609. doi: 10.1099/00207713-43-3-606, PMID: 8347518

[ref47] Patil P. P. Kumar S. Midha S. Gautam V. Patil P. B. (2018). Taxonogenomics reveal multiple novel genomospecies associated with clinical isolates of *Stenotrophomonas maltophilia*. Microb. Genom. 4:e000207. doi: 10.1099/mgen.0.000207, PMID: 30084764 PMC6159553

[ref48] Qin S. Li J. Chen H. H. Zhao G. Z. Zhu W. Y. Jiang C. L.. (2009). Isolation, diversity, and antimicrobial activity of rare actinobacteria from medicinal plants of tropical rain forests in Xishuangbanna, China. Appl. Environ. Microbiol. 75, 6176–6186. doi: 10.1128/AEM.01034-09, PMID: 19648362 PMC2753051

[ref49] Ratledge C. Dover L. G. (2000). Iron metabolism in pathogenic bacteria. Annu. Rev. Microbiol. 54, 881–941. doi: 10.1146/annurev.micro.54.1.881 11018148

[ref50] Saitou N. Nei M. (1987). The neighbor-joining method: a new method for reconstructing phylogenetic trees. Mol. Biol. Evol. 4, 406–425. doi: 10.1093/oxfordjournals.molbev.a040454, PMID: 3447015

[ref51] Sanchez-Castro I. Ruiz-Fresneda M. A. Bakkali M. Kampfer P. Glaeser S. P. Busse H. J.. (2017). *Stenotrophomonas bentonitica* sp. nov., isolated from bentonite formations. Int. J. Syst. Evol. Microbiol. 67, 2779–2786. doi: 10.1099/ijsem.0.002016, PMID: 28820086 PMC5817250

[ref52] Schwyn B. Neilands J. B. (1987). Universal chemical assay for the detection and determination of siderophores. Anal. Biochem. 160, 47–56. doi: 10.1016/0003-2697(87)90612-9, PMID: 2952030

[ref53] Shea C. M. McIntosh M. A. (1991). Nucleotide sequence and genetic organization of the ferric enterobactin transport system: homology to other periplasmic binding protein-dependent systems in Escherichia coli. Mol. Microbiol. 5, 1415–1428. doi: 10.1111/j.1365-2958.1991.tb00788.x, PMID: 1838574

[ref54] Staudenmaier H. Van-Hove B. Yaraghi Z. Braun V. (1989). Nucleotide sequences of the fecBCDE genes and locations of the proteins suggest a periplasmic-binding-protein-dependent transport mechanism for iron(III) dicitrate in Escherichia coli. J. Bacteriol. 171, 2626–2633. doi: 10.1128/jb.171.5.2626-2633.1989, PMID: 2651410 PMC209944

[ref55] Suckstorff I. Berg G. (2003). Evidence for dose-dependent effects on plant growth by Stenotrophomonas strains from different origins. J. Appl. Microbiol. 95, 656–663. doi: 10.1046/j.1365-2672.2003.02021.x, PMID: 12969277

[ref56] Swings J. P. P. Vos P. D. Mooter M. Ley J. D. (1983). Transfer of *pseudomonas maltophilia* Hugh 1981 to the genus *Xanthomonas* as *Xanthomonas maltophilia* (Hugh 1981) comb. nov. Int. J. Syst. Evol. Microbiol. 33, 409–413. doi: 10.1099/00207713-33-2-409

[ref57] Thomas C. M. Nielsen K. M. (2005). Mechanisms of, and barriers to, horizontal gene transfer between bacteria. Nat. Rev. Microbiol. 3, 711–721. doi: 10.1038/nrmicro1234 16138099

[ref58] Tullio L. D. Nakatani A. S. Gomes D. F. Ollero F. J. Megías M. Hungria M. (2019). Revealing the roles of y4wF and tidC genes in *rhizobium tropici* CIAT 899: biosynthesis of indolic compounds and impact on symbiotic properties. Arch. Microbiol. 201, 171–183. doi: 10.1007/s00203-018-1607-y, PMID: 30535938

[ref59] Ulrich K. Kube M. Becker R. Schneck V. Ulrich A. (2021). Genomic analysis of the endophytic *Stenotrophomonas* strain 169 reveals features related to plant-growth promotion and stress tolerance. Front. Microbiol. 12:687463. doi: 10.3389/fmicb.2021.687463, PMID: 34220780 PMC8245107

[ref60] Weber M. Schunemann W. Fuss J. Kampfer P. Lipski A. (2018). *Stenotrophomonas lactitubi* sp. nov. and *Stenotrophomonas indicatrix* sp. nov., isolated from surfaces with food contact. Int. J. Syst. Evol. Microbiol. 68, 1830–1838. doi: 10.1099/ijsem.0.002732, PMID: 29638210

[ref61] Wiener M. C. (2005). TonB-dependent outer membrane transport: going for Baroque? Curr. Opin. Struct. Biol. 15, 394–400. doi: 10.1016/j.sbi.2005.07.001, PMID: 16039843

[ref62] Wolf A. Fritze A. Hagemann M. Berg G. (2002). *Stenotrophomonas rhizophila* sp. nov., a novel plant-associated bacterium with antifungal properties. Int. J. Syst. Evol. Microbiol. 52, 1937–1944. doi: 10.1099/00207713-52-6-1937 12508851

[ref63] Yang H. C. Im W. T. Kang M. S. Shin D. Y. Lee S. T. (2006). *Stenotrophomonas koreensis* sp. nov., isolated from compost in South Korea. Int. J. Syst. Evol. Microbiol. 56, 81–84. doi: 10.1099/ijs.0.63826-0, PMID: 16403870

[ref64] Yoon S. H. Ha S. M. Kwon S. Lim J. Kim Y. Seo H.. (2017a). Introducing EzBioCloud: a taxonomically united database of 16S rRNA gene sequences and whole-genome assemblies. Int. J. Syst. Evol. Microbiol. 67, 1613–1617. doi: 10.1099/ijsem.0.001755, PMID: 28005526 PMC5563544

[ref65] Yoon S. H. Ha S. M. Lim J. Kwon S. Chun J. (2017b). A large-scale evaluation of algorithms to calculate average nucleotide identity. Antonie Van Leeuwenhoek 110, 1281–1286. doi: 10.1007/s10482-017-0844-4, PMID: 28204908

[ref66] Yuan L. J. Zhang Y. Q. Guan Y. Wei Y. Z. Li Q. P. Yu L. Y.. (2008). Saccharopolyspora antimicrobica sp. nov., an actinomycete from soil. Int. J. Syst. Evol. Microbiol. 58, 1180–1185. doi: 10.1099/ijs.0.65532-0, PMID: 18450710

