# Supplementary material for: Characterization of three Stenotrophomonas strains isolated from different ecosystems and proposal of Stenotrophomonas mori sp. nov. and Stenotrophomonas lacuserhaii sp. nov
Source: Front Microbiol. 2022 Dec 15;13:1056762. doi: 10.3389/fmicb.2022.1056762 (PMC9797726; doi:10.3389/fmicb.2022.1056762)
Supplement: Supplementary file 1 [file Data_Sheet_3.pdf]

Characterization of three *Stenotrophomonas* strains and proposal of *Stenotrophomonas mori* sp. nov., and *Stenotrophomonas lacuserhaii* sp. nov.

Yang Deng[1,2], Xue-Fei Han[1,2], Zhu-Ming Jiang[1,2], Li-Yan Yu[1], Yong Li[3], Yu-Qin Zhang[1,2*]

[1]Institute of Medicinal Biotechnology, Chinese Academy of Medical Sciences & Peking Union Medical College, Beijing 100050, P. R. China

[2]State Key Laboratory of Dao-di Herb, Beijing, 100700, P. R. China

[3]Faculty of Geosciences and Environmental Engineering, Southwest Jiaotong University, Chengdu 610031, PR China.

[*]Author for correspondence:

Yu-Qin Zhang

Tel: +86-10-83167110

Fax: +86-10-83167110

E-Mail: yzhang@imb.pumc.edu.cn

Keywords: ***Stenotrophomonas mori*, *Stenotrophomonas lacuserhaii*, genome, ANI, dDDH, IAA**

Abbreviations: **DPG, diphosphatidylglycerol; PG, phosphatidylglycerol; PE, phosphatidylethanolamine; ANI, average nucleotide identity; dDDH, digital DNA-DNA hybridization; IAA, indole-3-acetic acid; MALDI-TOF MS, matrix-assisted laser desorption/ionization time-of-flight mass spectrometry.**

Supplementary Figure S1. Transmission electron micrographs of motile cells of strains CPCC 101365^T and CPCC 101269^T.

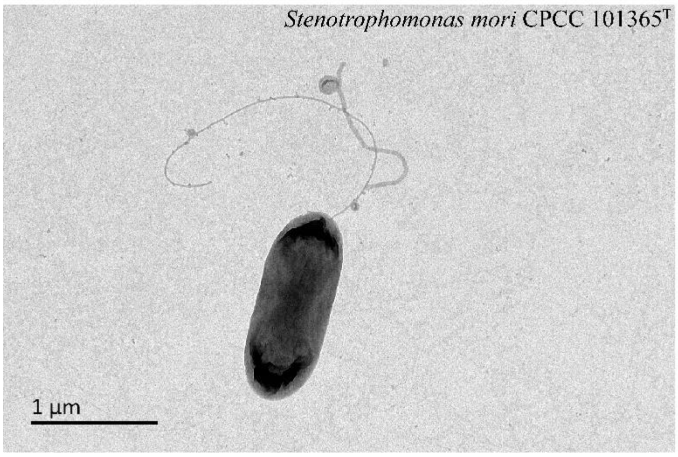

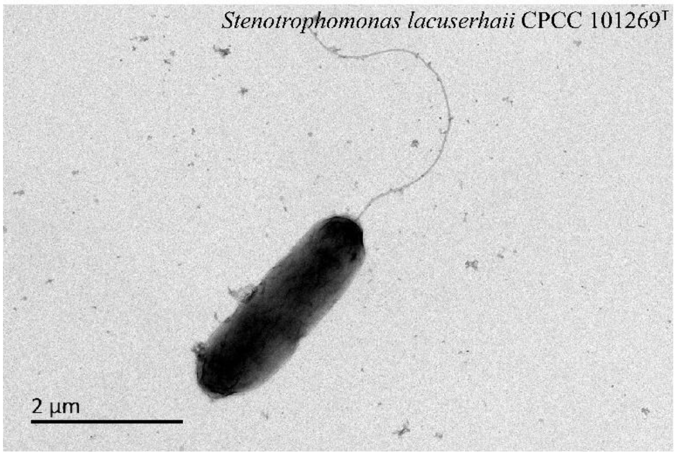

Supplementary Figure S2. Polar lipid profiles of strains CPCC 101365T, CPCC 101269T, and CPCC 101426 after separation by two-dimensional TLC.

(a) Detection by spraying with molybdatophosphoric acid reagent. (b) Detection by spraying with ninhydrin stain reagent. (c) Detection by spraying with molybdenum blue stain reagent. (d) Detection by spraying with p-anisaldehyde stain reagent.

DPG, diphosphatidylglycerol; PG, phosphatidylglycerol; PE, phosphatidylethanolamine; GPL, unidentified glycophospholipid; PL, unidentified phospholipid.

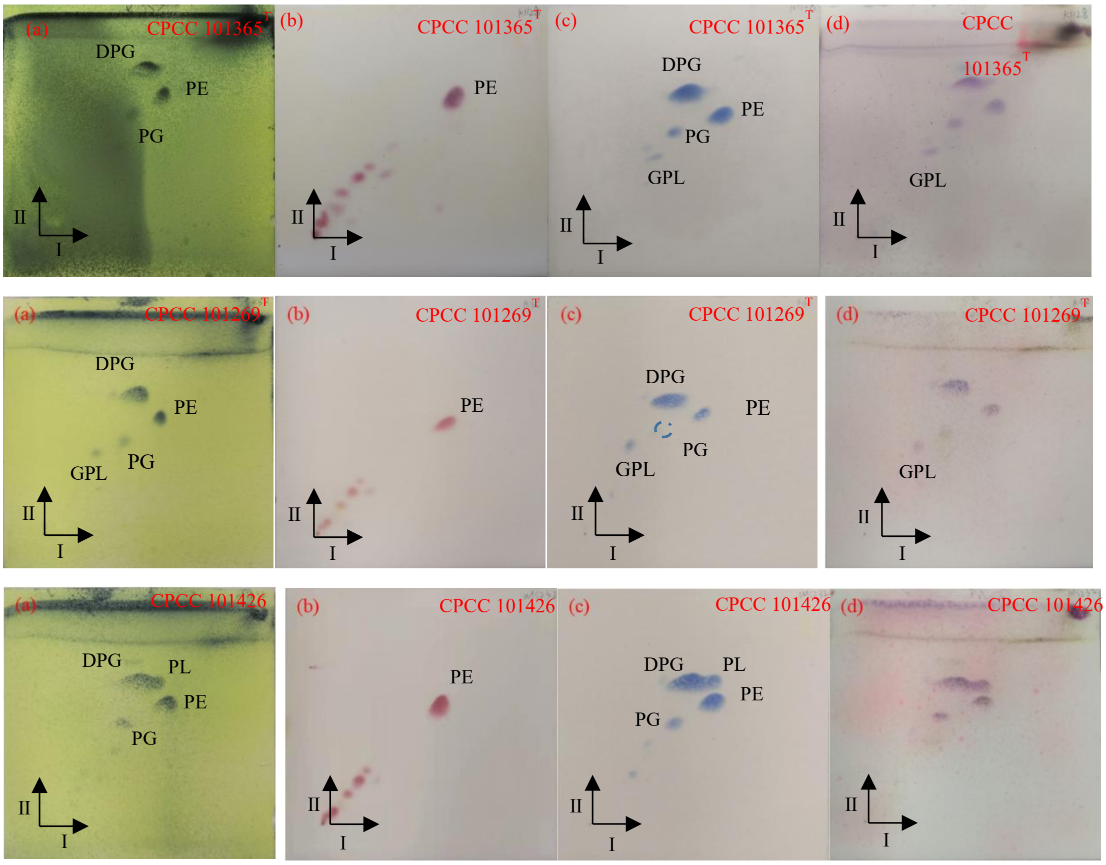

Supplementary Figure S3. Whole-genome phylogenetic tree generated for strains CPCC 101365^{T}, CPCC 101269^{T}, CPCC 101426, and other representatives of the family *Lysobacteraceae*.

The phylogenetic tree was inferred with the FastME algorithm using GBDP distances calculated from genome sequences. Branch lengths are scaled based on the GBDP distance formula d5. Numbers above branches show GBDP pseudo-bootstrap support values from 100 replicates.

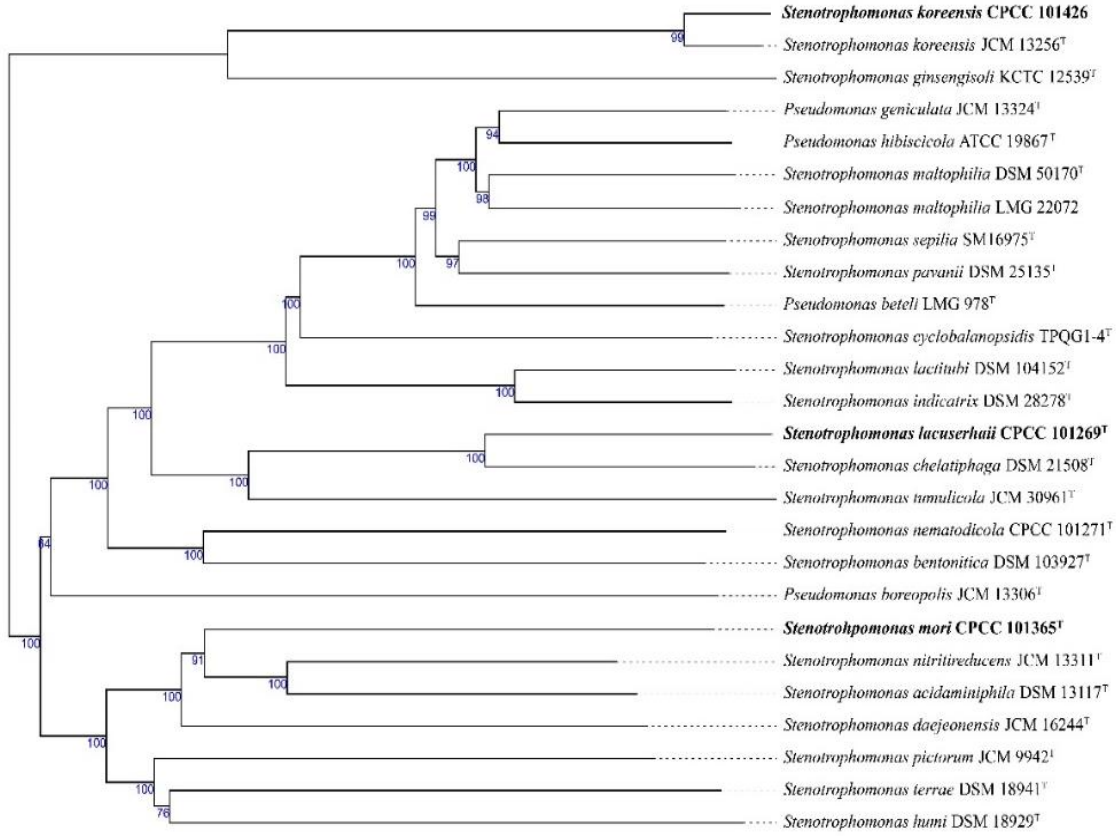

Supplementary Figure S4. MALDI-TOF MS spectra profiles of strains CPCC 101365^{T}, CPCC 101269^{T}, CPCC 101426, and closely related *Stenotrophomonas* strains.

The name of each strain was labeled on the upper right corner of each spectrum. (a) MALDI-TOF MS spectra profiles of strains CPCC 101365^{T}, CPCC 101269^{T}, CPCC 101426, and their closely related *Stenotrophomonas* strains; (b) MALDI-TOF MS profiles of strain CPCC 101365^{T} compared with *Stenotrophomonas acidaminiphila* DSM 13117^{T} and *Stenotrophomonas nitritireducens* JCM 13311^{T}, with the detail of peptide peaks of 6,429.9 Da and 11,254.6 Da in the spectra of strain CPCC 101365^{T}, *S. nitritireducens* JCM 13311^{T} and *S. acidaminiphila* DSM 13117^{T}; (c) MALDI-TOF MS profiles of strain CPCC 101269^{T} compared with *Stenotrophomonas chelatiphaga* DSM 21508^{T} and *Stenotrophomonas tumulicola* JCM 30961^{T}, with the detail of the peptide peak of 9,812.5 Da in the spectra of strain CPCC 101269^{T}, *Stenotrophomonas chelatiphaga* DSM 21508^{T} and *Stenotrophomonas tumulicola* JCM 30961^{T}.

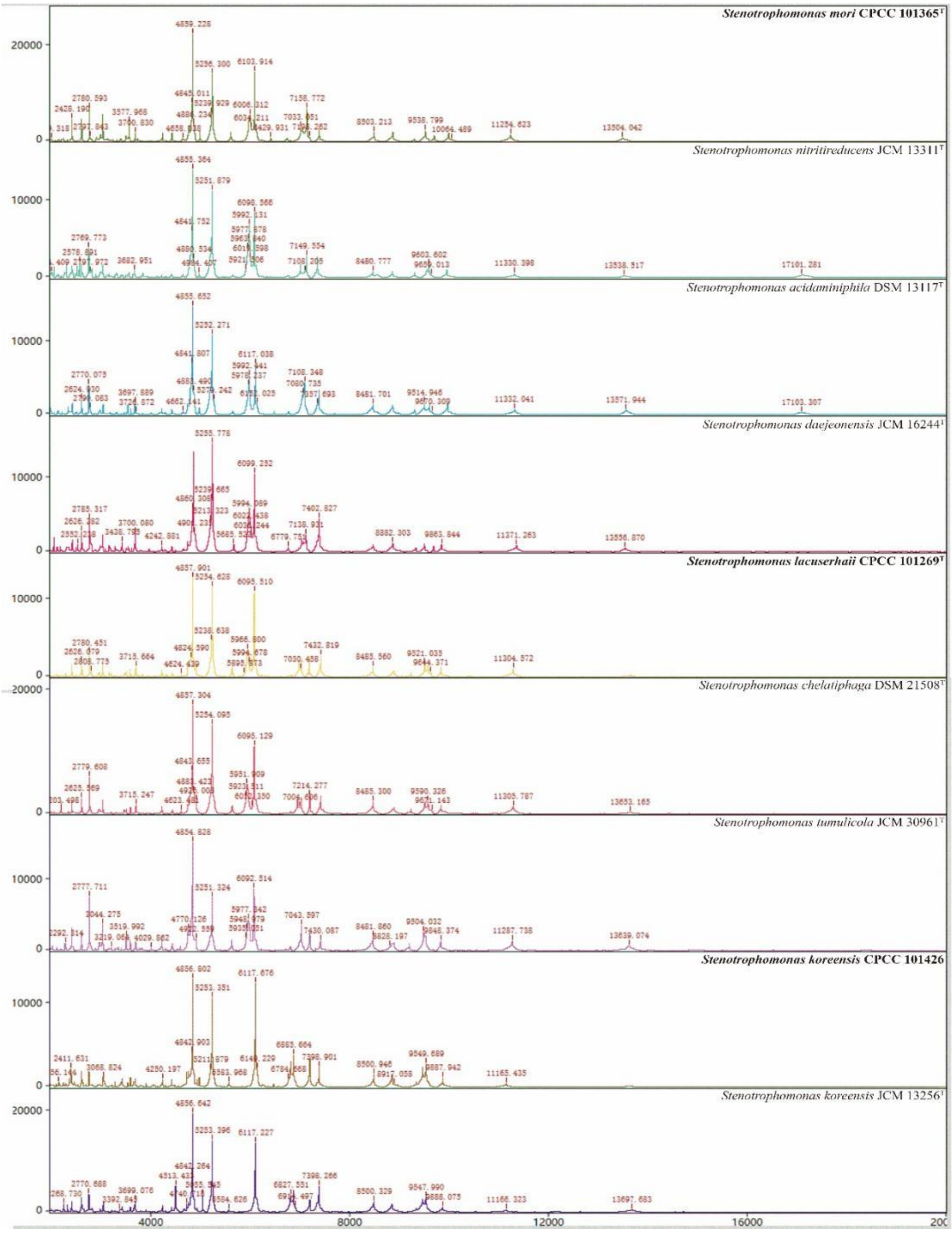

(a)

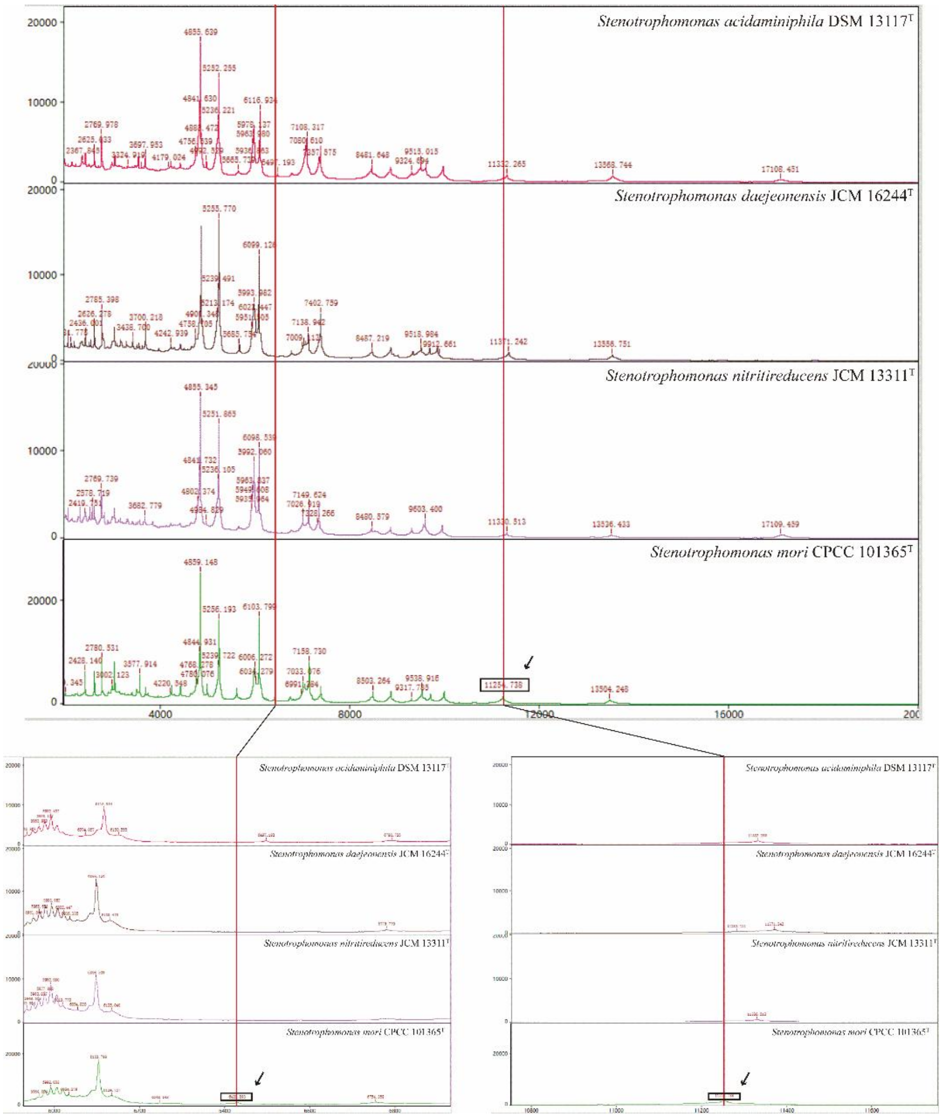
Stenotrophomonas acidaminiphila DSM 13117T
Stenotrophomonas daejeonensis JCM 16244T
Stenotrophomonas nitritireducens JCM 13311T
Stenotrophomonas mori CPCC 101365T
20000
10000
0
4000
8000
12000
16000
4855, 639
5252, 255
6116, 934
5236, 221
7105, 317
11332, 265
13568, 744
17108, 431
5255, 770
6099, 126
7402, 759
11371, 242
13556, 751
4855, 345
5251, 863
6098, 539
11330, 513
13536, 433
17109, 439
4859, 148
5256, 193
6103, 799
7158, 730
11254, 738
13504, 248

(b)

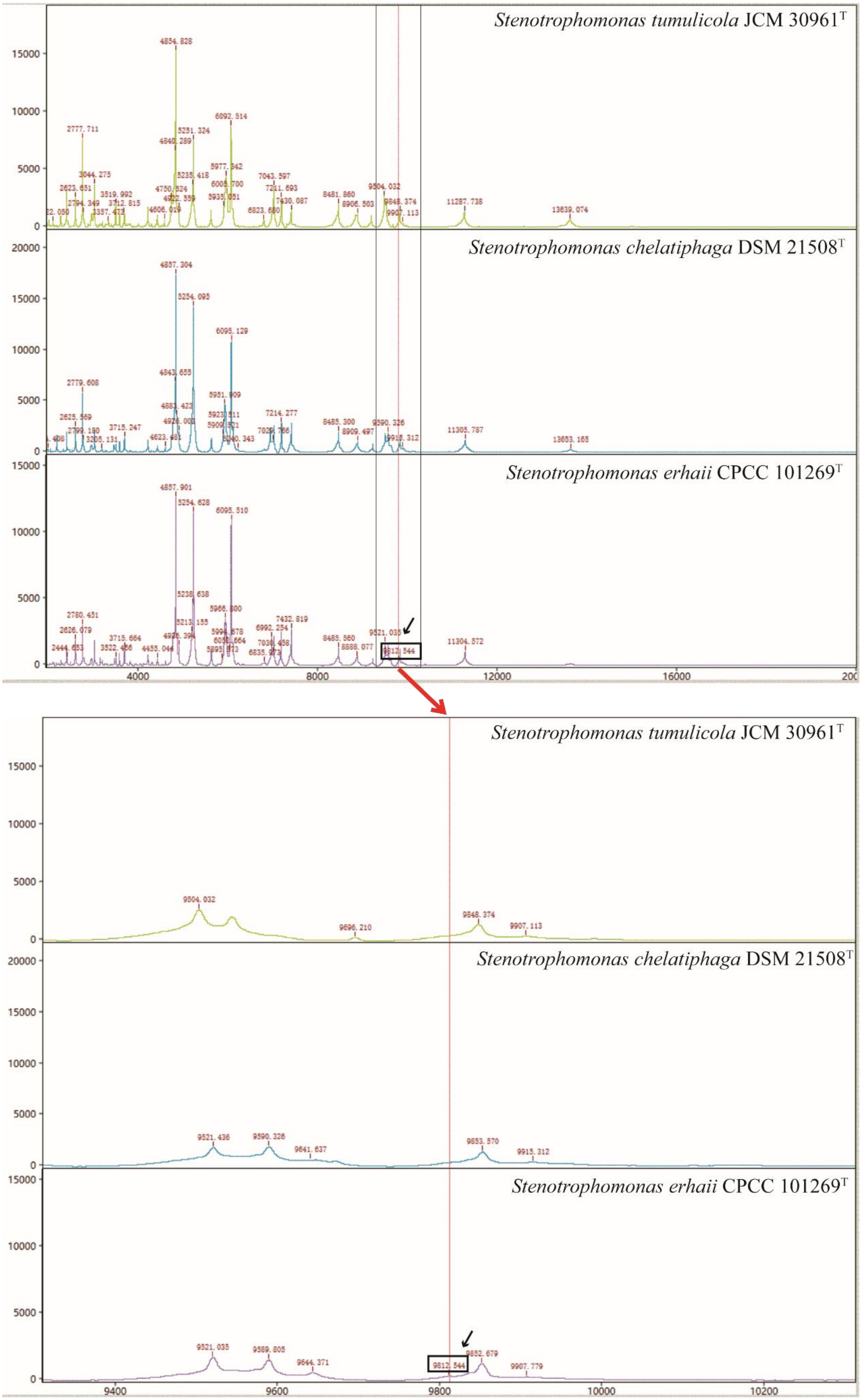
Stenotrophomonas tumulicola JCM 30961T
Stenotrophomonas chelatiphaga DSM 21508T
Stenotrophomonas erhaii CPCC 101269T
Stenotrophomonas tumulicola JCM 30961T
Stenotrophomonas chelatiphaga DSM 21508T
Stenotrophomonas erhaii CPCC 101269T

(c)

Supplementary Figure S5. Antibiotic resistance genes predicted from the genomes of CPCC 101365^T, CPCC 101269^T, CPCC 101426, *S. maltophilia* DSM 50170^T, and *S. maltophilia* K279a.

Strain 1, CPCC 101365^T; Strain 2, CPCC 101269^T; Strain 3, CPCC 101426; Strain 4, *S. maltophilia* DSM 50170^T; Strain 5, *S. maltophilia* K279a.

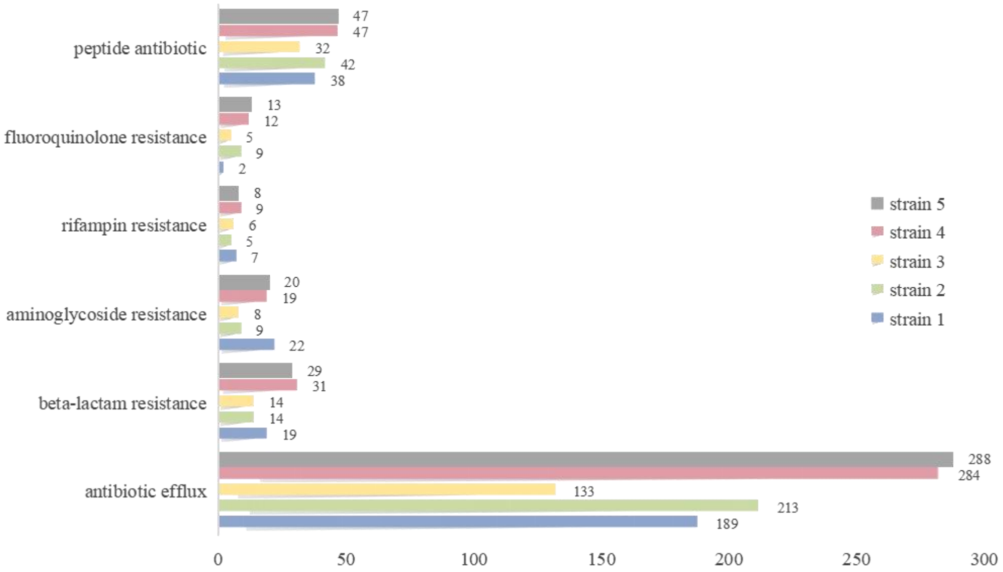

Supplementary Table S1. Cellular fatty acid profiles of strains CPCC 101365^T, CPCC 101269^T, CPCC 101426 and other closely related strains.

Strains: 1, CPCC 101365^T; 2, *Stenotrophomonas nitritireducens* JCM 13311^T; 3, *Stenotrophomonas acidaminiphila* DSM 13117^T. 4, *Stenotrophomonas daejeonensis* JCM 16244^T; 5, CPCC 101269^T; 6, *Stenotrophomonas chelatiphaga* DSM 21508^T; 7, *Stenotrophomonas tumulicola* JCM 30961^T; 8, CPCC 101426; 9, *Stenotrophomonas koreensis* JCM 13256^T. All data were generated in this study. -, not detected.

Fatty acids	1	2	3	4	5	6	7	8	9
**Straight-chain fatty acid**									
$C_{10:0}$	0.5	0.9	-	1.8	0.6	1.1	0.6	0.9	0.5
$C_{14:0}$	3.0	3.4	0.5	5.7	6.9	7.4	6.7	2.8	2.5
$C_{16:0}$	3.6	3.6	1.7	3.9	**12.2**	10.6	14.6	3.0	5.6
$C_{17:0}$		-	-	-	0.6	-	-	0.7	0.6
$C_{18:0}$		-	-	-	1.3	-	2.9	-	2.9
$C_{17:0}$ cyclo	0.8	1.7	0.9	-	1.6	-	2.5	3.0	1.9
$C_{15:1}\omega 6c$	-	-	-	-	-	-	-	0.5	-
$C_{15:1}\omega 8c$	-	-	-	-	-	-	-	1.6	1.2
$C_{16:1}\omega 9c$	0.9	0.6	0.6	-	1.7	1.6	1.8	-	-
$C_{17:1}\omega 8c$	1.0	-	-	-	-	-	-	1.1	0.8
$C_{18:1}\omega 9c$	-	-	-	-	0.6	-	1.0	-	-
**Iso-branched fatty acid**									
Iso-$C_{10:0}$	-	-	3.6	-	-	-	-	0.6	-
Iso-$C_{11:0}$	8.1	17.9	6.1	11.6	4.0	7.0	3.7	5.1	5.0
Iso-$C_{13:0}$	0.8	2.1	0.6	1.9	0.7	0.9	0.9	1.3	1.9
Iso-$C_{14:0}$	5.2	5.3	14.6	2.5	2.7	4.5	1.4	5.6	2.6
Iso-$C_{15:0}$	**36.7**	34.3	20.3	43.7	**27.4**	23.7	39.3	**40.6**	37.9
Iso-$C_{15:1}$ F	2.0	3.1	2.6	3.5	2.4	2.8	1.1	6.2	7.8
Iso-$C_{16:0}$	3.2	2.0	13.9	0.5	1.6	2.3	1.2	3.6	2.0
Iso-$C_{17:0}$	1.2	0.8	1.2	0.7	0.7	0.6	1.3	1.7	1.7
**Antesio-branched fatty acid**									
Antesio-$C_{11:0}$	0.6	1.1	-	0.6	-	0.6	-	0.6	0.6
Antesio-$C_{13:0}$	-	-	-	-	-	-	-	-	1.9
Antesio-$C_{14:0}$	-	-	-	-	-	-	-	-	2.6

Antesio-$C_{15:0}$	9.5	5.9	4.6	3.9	**16.6**	14.8	8.8	4.7	6.1
Antesio-$C_{17:0}$							-	-	0.5
**Hydroxy fatty acids**									
$C_{11:0}$ 3OH	0.5	0.5	-	-	-	-	-	0.9	0.7
$C_{12:0}$ 3OH	1.2	1.7	1.1	0.8	1.9	2.5	1.5	0.6	-
$C_{13:0}$ 2OH	1.1	1.1		0.5	0.9	1.5	0.5	-	-
Iso-$C_{11:0}$ 3OH	2.8	3.9	0.5	3.2	1.7	2.7	1.7	2.7	3.1
Iso-$C_{12:0}$ 3OH	1.7	1.8	5.8	0.5	-	1.0	-	0.7	-
Iso-$C_{13:0}$ 3OH	2.3	2.9	2.9	2.2	1.5	2.5	1.9	1.5	1.6
**Others**									
Sum In Feature 3 ($C_{16:1}\omega 7c$/$C_{16:1}\omega 6c$)	5.1	-	2.0	3.4	5.1	6.7	1.7	2.5	1.3
Sum In Feature 8 ($C_{18:1}\omega 7c$/$C_{18:1}\omega 6c$)	-	-	-	-	0.8	-	1.0	-	-
Sum In Feature 9 (iso- $C_{17:1}\omega 9c$/$C_{16:0}$ 10-methyl)	5.3	2.5	10.2	4.4	3.3	1.9	1.8	4.2	5.4

Supplementary Table S2. Pairwise comparison of the 16S rRNA gene sequences of strains CPCC 101365^{T}, CPCC 101269^{T}, CPCC 101426, and other closely related strains.

1, CPCC 101365^{T}; 2, *Stenotrophomonas nitritireducens* JCM 13311^{T}; 3, *Stenotrophomonas acidaminiphila* DSM 13117^{T}; 4, *Stenotrophomonas daejeonensis* JCM 16244^{T}; 5, CPCC 101269^{T}; 6, *Stenotrophomonas chelatiphaga* DSM 21508^{T}; 7, *Stenotrophomonas tumulicola* JCM 30961^{T}; 8, CPCC 101426; 9, *Stenotrophomonas koreensis* JCM 13256^{T}; 10, *Stenotrophomonas ginsengisoli* KCTC 12539^{T}.

16S rRNA gene sequence similarities (%) > 98.65 % were highlighted in red.

Species	1	2	3	4	5	6	7	8	9	10
1	100.0									
2	99.1	100.0								
3	98.8	98.7	100.0							
4	98.0	97.0	98.4	100.0						
5	97.3	97.3	97.3	97.1	100.0					
6	97.4	97.3	97.1	96.6	99.9	100.0				
7	97.8	97.4	97.3	96.6	99.4	99.4	100.0			
8	97.1	97.1	97.2	96.3	97.3	97.4	97.6	100.0		
9	97.2	97.2	97.3	96.4	97.6	97.5	97.6	99.8	100.0	
10	96.7	97.1	96.7	96.7	97.6	97.5	97.6	98.3	98.4	100.0

Supplementary Table S3. Genome features of strains CPCC 101365^{T}, CPCC 101269^{T}, CPCC 101426, and other closely related strains.

Strains: 1, CPCC 101365^{T}; 2, *Stenotrophomonas nitritireducens* JCM 13311^{T}; 3. *Stenotrophomonas acidaminiphila* DSM 13117^{T}; 4, *Stenotrophomonas daejeonensis* JCM 16244^{T}; 5, CPCC 101269^{T}; 6, *Stenotrophomonas chelatiphaga* DSM 21508^{T}; 7, *Stenotrophomonas tumulicola* JCM 30961^{T}; 8, CPCC 101426; 9, *Stenotrophomonas koreensis* JCM 13256^{T}.

*Data were derived from this study except: [a], Finkmann et al., 2000; [b], Assih et al., 2002; [c], Lee et al., 2011; [d], Kaparullina et al., 2009; [e], Handa et al., 2016; [f], Yang et al., 2006.

**Characteristic**	**1**	**2**	**3**	**4**	**5**	**6**	**7**	**8**	**9**
Isolation source	Mulberry root	Ammonia-supplied	Upflow anaerobic	Sewage water sample [c]	Fresh water reservoir	Sewage sludge [d]	Major contaminant of	Desert sandy soil	Compost [f]

		biofilters [a]	sludge blanket [b]				stone chamber interior [e]		
No. of contigs	11	95	126	124	27	28	26	16	58
N50 Length (bp)	468,441	167,790	71,728	46,611	381,161	227,000	313,801	521,188	188,329
Genome size (Mbp)	3.4	4.0	3.9	3.3	4.0	4.0	4.3	3.1	3.0
G+C (%)	70.2	68.3	68.8	68.6	66.4	66.9	65.6	66.2	66.1
No. of CDS	2,860	3,490	3,554	2,959	3,439	3,378	3,774	2,706	2,598
DDBJ/EMBL/Genbnk accession number of draft genome	JAIKTS000000000	LDJG00000000	LDJO00000000	LDJP00000000	JACHQY000000000	LDJK00000000	JACGXS000000000	JACIUV000000000	LDJH00000000
GenBank/RefSeq assembly accession number	GCA_023556335.1	GCA_001431425.1	GCA_001431595.1	GCA_001431505.1	GCA_014596295.1	GCA_001431535.1	GCA_014117215.1	GCA_014145815.1	GCA_001431525.1

Supplementary Table S4. Average nucleotide identity (ANI) (%) values between strains CPCC 101365^{T}, CPCC 101269^{T}, CPCC 101426, and other *Stenotrophomonas* type strains.

Strains: 1, CPCC 101365^{T}; 2, CPCC 101269^{T}; 3, CPCC 101426; 4, *S. acidaminiphila* DSM 13117^{T}; 5, *S. bentonitica* DSM 103927^{T}; 6, *S. chelatiphaga* DSM 21508^{T}; 7, *S. daejeonensis* JCM 16244^{T}; 8, *S. ginsengisoli* KCTC 12539^{T}; 9, *S. humi* DSM 18929^{T}; 10, *S. indicatrix* DSM 28278^{T}; 11, *S. koreensis* JCM 13256^{T}; 12, *S. lactitubi* DSM 104152^{T}; 13, *S. maltophilia* DSM 50170^{T}; 14, *S. nitritireducens* JCM 13311^{T}; 15, *S. pavanii* DSM 25135^{T}; 16, *S. pictorum* JCM 9942^{T}; 17, *S. rhizophila* JCM 13333^{T}; 18, *S. terrae* DSM 18941^{T}; 19, *S. tumulicola* JCM 30961^{T}.

ANI values >95 % were highlighted in red.

Reference strains	Accession numbers of genomic data	ANI value (%)																		
		1	2	3	4	5	6	7	8	9	10	11	12	13	14	15	16	17	18	19
CPCC 101365^{T}	JAIKTS000000000	100.0																		
CPCC 101269^{T}	JACHQY000000000	79.2	100.0																	
CPCC 101426	JACIUV000000000	77.9	76.6	100.0																
S. acidaminiphila DSM 13117^{T}	JAMHDZ000000000	86.2	79.4	78.2	100.0															
S. bentonitica DSM 103927^{T}	JAAZUH000000000	80.0	81.3	77.0	80.5	100.0														
S. chelatiphaga DSM 21508^{T}	LDJK00000000	79.4	91.8	76.6	79.9	81.4	100.0													
S. daejeonensis	LDJP00000000	85.1	79.6	78.1	86.4	80.7	79.8	100.0												

JCM 16244^{T}																		
S. ginsengisoli KCTC 12539^{T}	LDJM00000000	77.4	76.5	84.0	77.9	77.0	76.9	77.9	100.0									
S. humi DSM 18929^{T}	LDJI00000000	81.3	78.5	76.5	82.2	79.4	78.5	82.4	76.6	100.0								
S. indicatrix DSM 28278^{T}	PEJS00000000	80.0	82.2	76.6	80.0	81.5	82.3	80.3	76.8	78.9	100.0							
S. koreensis JCM 13256^{T}	LDJH00000000	77.8	76.5	97.7	78.0	76.9	76.7	77.9	84.6	76.5	76.6	100.0						
S. lactitubi DSM 104152^{T}	PHQX00000000	79.4	82.1	76.7	79.8	81.5	82.3	80.0	76.6	78.5	93.5	76.4	100.0					
S. maltophilia DSM 50170^{T}	BCUI00000000	79.8	82.2	77.1	80.3	81.8	82.3	80.4	76.8	78.9	86.9	76.9	87.0	100.0				
S. nitritireducens JCM 13311^{T}	LDJG00000000	86.2	79.7	78.1	90.0	81.1	79.9	87.0	77.9	82.5	80.3	78.0	80.5	80.4	100.0			
S. pavanii DSM 25135^{T}	LDJN00000000	79.8	82.2	77.1	80.3	81.8	82.2	80.5	77.0	78.7	87.0	76.7	87.1	91.3	80.6	100.0		
S. pictorum JCM 9942^{T}	LLXS00000000	82.4	78.7	77.5	83.2	79.9	78.8	83.1	77.4	83.0	79.1	77.6	79.0	79.6	83.7	79.2	100.0	
S. rhizophila JCM 13333^{T}	CP007597	80.0	81.4	77.0	80.5	85.6	81.4	80.6	76.9	79.5	81.7	77.0	81.7	81.6	80.6	81.6	79.8	100.0

S. terrae DSM 18941^T	LDJJ00000000	81.4	78.7	71.0	82.3	79.3	78.4	82.4	77.0	83.6	78.8	77.2	78.6	78.8	82.6	79.1	83.2	79.3	100.0	
S. tumulicola JCM 30961^T	JACGXS000000000	78.8	84.5	76.0	79.3	80.9	84.7	79.4	76.4	77.9	81.9	76.1	81.8	82.1	79.3	82.0	78.4	80.9	78.0	100.0

Supplementary Table S5. Digital DNA–DNA hybridization (dDDH) (%) values between strains CPCC 101365^{T}, CPCC 101269^{T}, CPCC 101426, and other *Stenotrophomonas* type strains.

Strains: 1, CPCC 101365^{T}; 2, CPCC 101269^{T}; 3, CPCC 101426; 4, *S. acidaminiphila* DSM 13117^{T}; 5, *S. bentonitica* DSM 103927^{T}; 6, *S. chelatiphaga* DSM 21508^{T}; 7, *S. daejeonensis* JCM 16244^{T}; 8, *S. ginsengisoli* KCTC 12539^{T}; 9, *S. humi* DSM 18929^{T}; 10, *S. indicatrix* DSM 28278^{T}; 11, *S. koreensis* JCM 13256^{T}; 12, *S. lactitubi* DSM 104152^{T}; 13, *S. maltophilia* DSM 50170^{T}; 14, *S. nitritireducens* JCM 13311^{T}; 15, *S. pavanii* DSM 25135^{T}; 16, *S. pictorum* JCM 9942^{T}; 17, *S. rhizophila* JCM 13333^{T}; 18, *S. terrae* DSM 18941^{T}; 19, *S. tumulicola* JCM 30961^{T}.

dDDH values > 70 % were highlighted in red.

Reference strains	Accession numbers of genomic data	dDDH value (%)																		
		1	2	3	4	5	6	7	8	9	10	11	12	13	14	15	16	17	18	19
CPCC 101365^{T}	JAIKTS000000000	100.0																		
CPCC 101269^{T}	JACHQY000000000	22.5	100.0																	
CPCC 101426	JACIUV000000000	21.7	20.4	100.0																
S. acidaminiphila DSM 13117^{T}	JAMHDZ000000000	30.7	22.6	21.6	100.0															
S. bentonitica DSM 103927^{T}	JAAZUH000000000	23.0	24.1	20.6	23.6	100.0														
S. chelatiphaga DSM 21508^{T}	LDJK00000000	22.6	45.6	20.6	24.8	24.2	100.0													
S. daejeonensis	LDJP00000000	29.1	23.0	21.9	31.5	24.0	23.3	100.0												

JCM 16244^{T}																		
S. ginsengisoli KCTC 12539^{T}	LDJM00000000	21.1	20.6	27.2	21.2	20.7	20.7	21.7	100.0									
S. humi DSM 18929^{T}	LDJI00000000	24.8	22.2	21.0	25.8	22.6	22.1	26.0	21.1	100.0								
S. indicatrix DSM 28278^{T}	PEJS00000000	22.9	25.2	20.9	23.3	23.3	25.6	23.5	21.0	22.5	100.0							
S. koreensis JCM 13256^{T}	LDJH00000000	21.6	20.5	78.3	21.7	20.6	20.6	22.1	27.4	21.2	20.9	100.0						
S. lactitubi DSM 104152^{T}	PHQX00000000	22.8	25.1	21.0	23.1	24.9	25.5	23.6	20.7	22.3	52.5	21.0	100.0					
S. maltophilia DSM 50170^{T}	BCUI00000000	23.3	25.2	21.4	23.7	25.0	25.5	24.1	21.3	21.3	32.0	21.3	32.3	100.0				
S. nitritireducens JCM 13311^{T}	LDJG00000000	31.3	23.0	22.0	39.9	24.5	23.4	32.3	21.8	26.3	23.5	22.0	23.6	24.1	100.0			
S. pavanii DSM 25135^{T}	LDJN00000000	23.2	25.3	20.8	23.7	25.1	25.6	23.8	21.2	22.8	32.4	21.0	32.4	43.0	23.9	100.0		
S. pictorum JCM 9942^{T}	LLXS00000000	25.2	21.8	21.0	26.3	22.9	22.0	26.5	20.9	26.1	22.4	21.0	22.2	23.0	27.4	22.7	100.0	
S. rhizophila JCM 13333^{T}	CP007597	23.3	24.5	21.0	23.7	29.9	24.5	24.2	21.1	22.8	24.8	21.1	24.5	24.7	23.9	24.8	23.0	100.0

S. terrae DSM 18941T	LDJJ00000000	24.7	21.3	21.2	25.4	22.5	22.3	26.2	21.2	21.2	22.7	21.3	22.7	22.8	26.1	22.7	26.7	23.2	100.0	
S. tumulicola JCM 30961T	JACGXS000000000	22.6	28.2	20.6	22.4	24.0	28.7	23.0	20.5	22.0	25.1	20.6	25.1	25.5	23.0	25.3	22.0	24.0	22.0	100.0

Supplementary Table S6. Genetic features predicted from the genomes of strains CPCC 101365^T, CPCC 101269^T, and CPCC 101426 that may be potentially involved in plant growth-promotion and environmental stress tolerance. -, not detected.

Gene	Enzyme code	Product	CPCC 101365^T	CPCC 101269^T	CPCC 101426
Biosynthesis of indole-3-acetic acid					
trpE	EC 4.1.3.27	Anthranilate synthase component II	K5L01_RS12200	H4O12_RS06550	H4O09_RS01845
trpG	EC 4.1.3.27	Anthranilate synthase component I	K5L01_RS12210	H4O12_RS06555	H4O09_RS01850
trpD	EC 2.4.2.18	Anthranilate phosphoribosyltransferase	K5L01_RS12185	H4O12_RS06545	H4O09_RS01835
trpF	EC 5.3.1.24	Phosphoribosylanthranilate isomerase	K5L01_RS05645	H4O12_RS13755	H4O09_RS07325
trpC	EC 4.1.1.48	Indole-3-glycerol phosphate synthase	K5L01_RS12180	H4O12_RS06540	H4O09_RS01830
trpA	EC 4.2.1.20	Tryptophan synthase subunit alpha	K5L01_RS05625	H4O12_RS13770	H4O09_RS07280
trpB	EC 4.2.1.20	Tryptophan synthase subunit beta	K5L01_RS05635	H4O12_RS13765	H4O09_RS07290
	EC 1.2.7.8	Indolepyruvate ferredoxin oxidoreductase	K5L01_RS14095	H4O12_RS08805	-
Production and uptake of siderophores					
aroA	EC 2.5.1.19	3-phosphoshikimate 1-carboxyvinyltransferase	K5L01_RS04530	H4O12_RS02960	H4O09_RS05170
aroC	EC 4.2.3.5	Chorismate synthase	K5L01_RS05675	H4O12_RS13725	H4O09_RS10235
aroQ	EC 5.4.99.5	Gamma subclass chorismate mutase	-	H4O12_RS15100	-
pheA	EC 4.2.1.51	Prephenate dehydratase	K5L01_RS04535	H4O12_RS02955	H4O09_RS05165
tonB		TonB-dependent siderophore receptor	K5L01_RS06265, K5L01_RS09170, K5L01_RS09675, K5L01_RS04030	H4O12_RS03035, H4O12_RS03085, H4O12_RS03135, H4O12_RS04010, H4O12_RS04930, H4O12_RS08610, H4O12_RS00795	-
		TonB family protein	K5L01_RS04520, K5L01_RS08370	-	H4O09_RS12115, H4O09_RS14025
exbB		TonB-system energizer	K5L01_RS09450	H4O12_RS09260	H4O09_RS03300
exbD		Biopolymer transporter	K5L01_RS09455, K5L01_RS09460, K5L01_RS09455	H4O12_RS01135, H4O12_RS09250, H4O12_RS09255	H4O09_RS07115, H4O09_RS03290, H4O09_RS03295
tolA		Cell envelope integrity protein	K5L01_RS01620	H4O12_RS11265	H4O09_RS11665
tolQ		MotA/TolQ/ExbB proton channel family protein	K5L01_RS05475	H4O12_RS17900	H4O09_RS07120
feoB		Ferrous iron transporter	K5L01_RS03155	H4O12_RS16055	H4O09_RS06280

ahpC	Peroxiredoxin	-	H4O12_RS12965	H4O09_RS00175
fiu	Catecholate siderophore receptor	-	H4O12_RS03715	-
bfr	Bacterioferritin	-	H4O12_RS06475, H4O12_RS00590	H4O09_RS01770
fecR	periplasmic ferric-dicitrate binding protein	K5L01_RS01055, K5L01_RS02360, K5L01_RS05375, K5L01_RS12910	-	H4O09_RS10730

Supplementary Table S7. Genes related to heat shock and suicide mechanisms predicted from the genomes of strains CPCC 101365^T, CPCC 101269^T, CPCC 101442, *S. maltophilia* K279a, and *S. maltophilia* DSM 50170^T.

Gene	Product	Biological role	CPCC 101365^T	CPCC 101269^T	CPCC 101426	*S. maltophilia* K279a	*S. maltophilia* DSM 50170^T
rpoH	Sigma 32	Heat shock sigma factor	K5L01_RS12545	H4O12_RS06290	H4O09_RS09420	SMLT_RS20270	CKW06_RS21085
dnaJ	Chaperone DnaJ	Stress response	K5L01_RS02800	H4O12_RS02310	H4O09_RS04855	SMLT_RS09635	CKW06_RS10350
dnaK	Chaperone DnaK	Stress response	K5L01_RS02795	H4O12_RS02305	H4O09_RS04850	SMLT_RS09630	CKW06_RS10345
grpE	Chaperone complex protein	Prevents the aggregation of stress-denatured proteins	K5L01_RS02790	H4O12_RS02300	H4O09_RS04845	SMLT_RS09625	CKW06_RS10340
groL	Chaperonin GroEL	Heat shock chaperone	K5L01_RS09590	H4O12_RS06075	H4O09_RS01565	SMLT_RS20060	CKW06_RS20875
htpG	Heat shock chaperone	Heat shock response	K5L01_RS03590	H4O12_RS01760	H4O09_RS05765	SMLT_RS08735	CKW06_RS09760
clpA	ATPase with chaperone activity	Stress response	K5L01_RS04170	H4O12_RS04830	H4O09_RS13075	SMLT_RS11235	CKW06_RS11950
hslV	Protease	Degradation of abnormal/stress-denatured proteins	K5L01_RS10195	H4O12_RS14930	H4O09_RS09155	SMLT_RS19390	CKW06_RS20190
htpX	Protease	Degradation of abnormal/stress-denatured proteins due to heat shock	K5L01_RS05205	H4O12_RS14930	H4O09_RS13225	SMLT_RS15810	CKW06_RS16180

lon	Lon Protease	Degradation of abnormal/stress-denatured proteins	K5L01_RS07160	H4O12_RS11945	H4O09_RS14160	SMLT_RS04765	CKW06_RS04660
	Killer protein	Suicide mechanism	K5L01_RS05285	H4O12_RS11850	-	SMLT_RS06410, SMLT_RS17165, SMLT_RS20265, SMLT_RS00305, SMLT_RS01585	CKW06_RS17925, CKW06_RS21080, CKW06_RS00205, CKW06_RS01455
	Entericidin EcnAB	Suicide mechanism	K5L01_RS08140, K5L01_RS08145	H4O12_RS08170, H4O12_RS08175	H4O09_RS10945, H4O09_RS10950	SMLT_RS01620	CKW06_RS17925
